# Biodegradable Grubbs-Loaded Artificial Organelles for Endosomal Ring-Closing Metathesis

**DOI:** 10.1021/acs.biomac.3c00487

**Published:** 2023-08-17

**Authors:** Roy A. J. F. Oerlemans, Jingxin Shao, Marleen H. M. E. van Stevendaal, Hanglong Wu, Tania Patiño Padial, Loai K. E. A. Abdelmohsen, Jan C. M. van Hest

**Affiliations:** Bio-Organic Chemistry, Institute for Complex Molecular Systems (ICMS), Eindhoven University of Technology, 5600 MB Eindhoven, The Netherlands

## Abstract

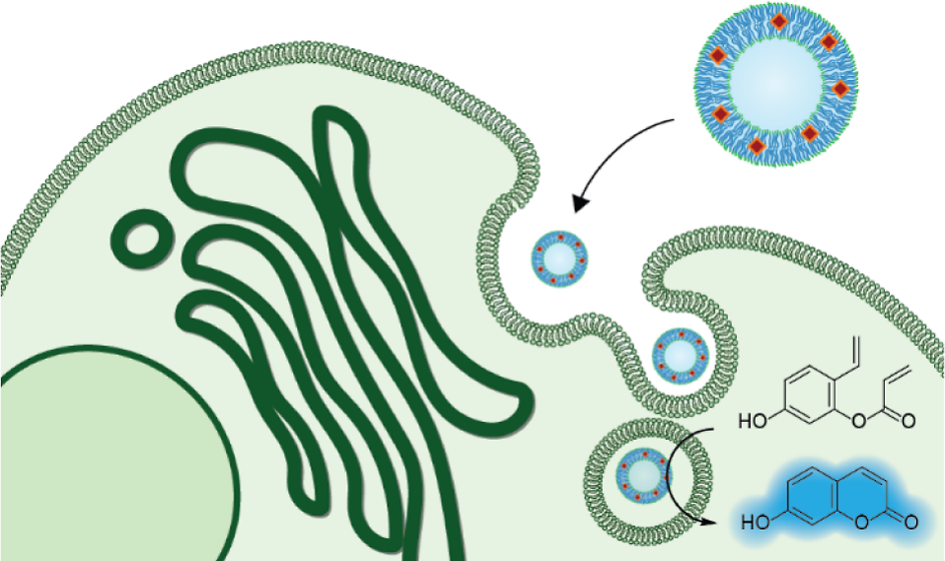

The application of transition-metal catalysts in living cells presents a promising approach to facilitate reactions that otherwise would not occur in nature. However, the usage of metal complexes is often restricted by their limited biocompatibility, toxicity, and susceptibility to inactivation and loss of activity by the cell’s defensive mechanisms. This is especially relevant for ruthenium-mediated reactions, such as ring-closing metathesis. In order to address these issues, we have incorporated the second-generation Hoveyda–Grubbs catalyst (HGII) into polymeric vesicles (polymersomes), which were composed of biodegradable poly(ethylene glycol)-*b*-poly(caprolactone-*g*-trimethylene carbonate) [PEG-*b*-P(CL-*g*-TMC)] block copolymers. The catalyst was either covalently or non-covalently introduced into the polymersome membrane. These polymersomes were able to act as artificial organelles that promote endosomal ring-closing metathesis for the intracellular generation of a fluorescent dye. This is the first example of the use of a polymersome-based artificial organelle with an active ruthenium catalyst for carbon–carbon bond formation.

## Introduction

In the past two decades, researchers have been exploring the prospect of using organometallic catalysis within biological systems.^1,2^ This approach has led to a wide range of potential abiotic reactions that can be introduced to cells, such as deprotections (typically alloc, poc, allyl, or propargyl), cycloadditions, and cross-couplings. This methodology is of great interest from a research perspective as it enables bio-orthogonal and targeted labeling of cellular components, for example. Furthermore, it shows potential in the biomedical field, for example in prodrug therapy, where the catalyst can synthesize therapeutic agents in the targeted cells.^3,4^

A long sought-after transition-metal-catalyzed reaction for intracellular applications is metathesis, a bio-orthogonal reaction that forms carbon–carbon bonds. Metathesis reactions can be catalyzed by the second-generation Hoveyda–Grubbs catalyst (HGII), which is a ruthenium-based complex, with a high degree of stability and resistance to both oxygen and moisture. However, similar to many other transition metals, HGII is highly toxic to cells, as demonstrated by their chemotherapeutic properties.^5,6^ Furthermore, living cells have evolved to neutralize foreign components to protect themselves which, in turn, leads to a hostile environment for organometallic catalysts. Deactivation in cells is mainly caused by thiolates, which, as soft Lewis bases, are apt to complex with soft Lewis acidic transition metals.^7^ Especially the thiol-containing peptide glutathione (GSH) is a key player in the cell’s redox balance, protecting it from heavy metal poisoning and metal-induced oxidative stress.^8^ Therefore, organometallic catalysts in cells often show activity only for short time spans, leading to low reaction yields. In consequence, highly sensitive techniques, such as those based on fluorescence, which require minimal product formation, are often utilized as readouts to evaluate activity.

Researchers have previously reported approaches to limit catalyst poisoning. For example, Ward and co-workers directed a ruthenium complex to the periplasm of bacteria, where GSH levels are considerably lower.^9^ Another strategy involved surrounding the catalyst with a negative charge to prevent glutathione from interacting.^10^ Both of these elegant solutions involved immobilization of a ruthenium catalyst in a confined space, in these cases the pocket of an artificial metalloprotein, a strategy also applied previously for a copper catalyst.^11^ However, these examples face challenges that make their general applicability rather difficult. In the first example, the cells had to be genetically engineered to express the host protein that could accommodate the modified catalyst. In the second example, the artificial metalloprotein was assembled first, but upon addition to cells, it remained inconclusive whether it was internalized in or associated with the cell surface. Nanotechnological approaches can be useful to provide a more generic approach for catalyst uptake and protection.^12^ Various nano- and micro-sized platforms have been investigated as vehicles for organometallic catalysts. Single-chain nanoparticles,^13^ metal–organic frameworks,^14,15^ silica nanoparticles,^16^ micelles,^17^ and coated metal^18,19^ and polystyrene^20^ nanoparticles have all been shown to transport transition metals inside living cells and locally promote abiotic conversions. Their biomedical application potential is, however, limited due to their non-degradable nature, and they have not been employed yet for the internalization of HGII.

**Figure 1:**
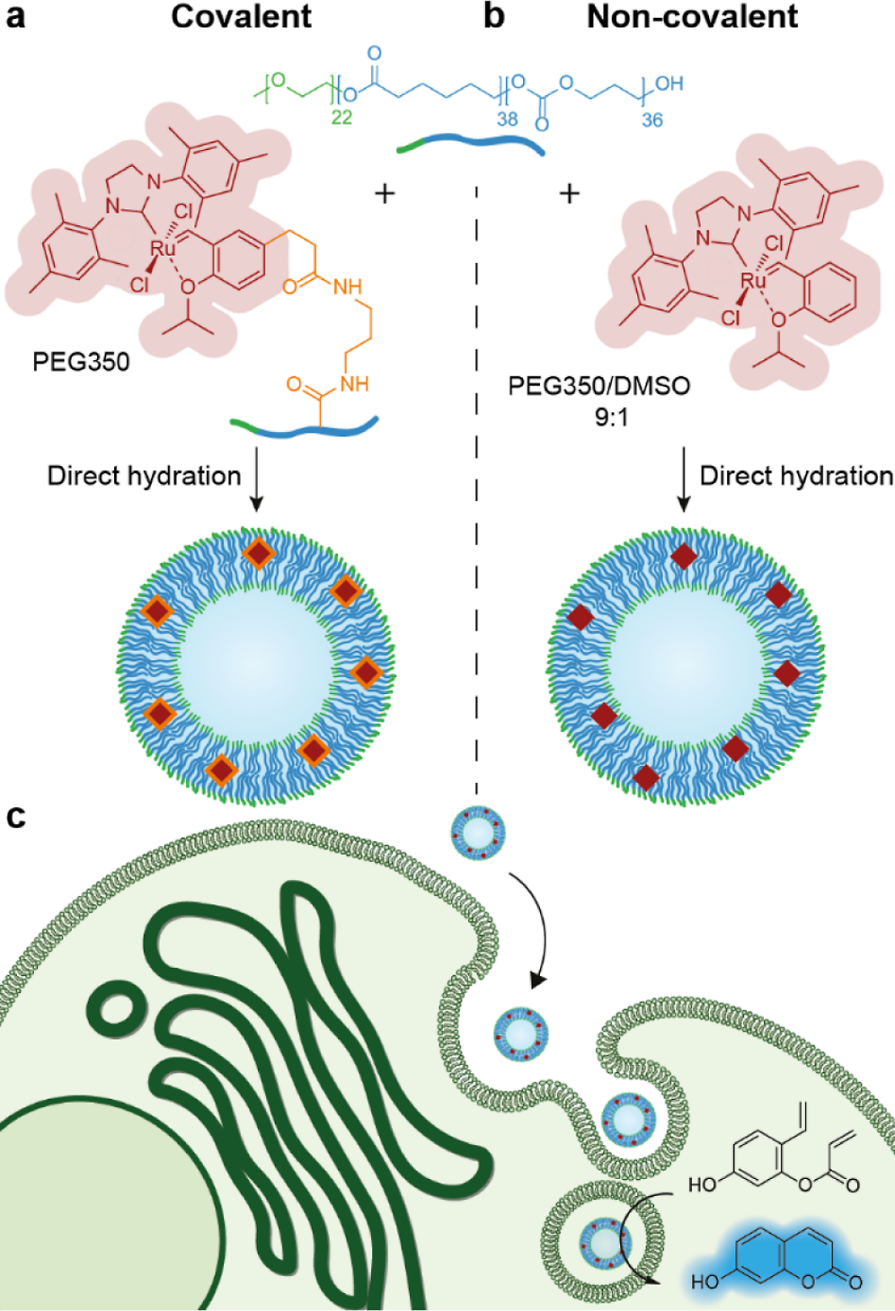
Schematic overview of the two approaches used to form Ru-loaded polymersome-based artificial organelles, which promote metathesis after cellular uptake. (a) Copolymer with the covalently bound HGII catalyst, dissolved in oligo(ethylene glycol) (PEG350), self-assembles into catalytic polymeric vesicles upon direct hydration using PBS. (b) A mixture of free HGII catalyst and copolymer is dissolved in a mixture of PEG350 and DMSO; upon self-assembly, the catalyst is embedded in the polymersome membrane by hydrophobic interactions. (c) Both approaches lead to polymersome-based artificial organelles, which are internalized by living cells to locally produce a fluorescent product via ring-closing metathesis.

We have recently reported the intracellular activity of polymeric vesicles (polymersomes) with an integrated copper catalyst.^21^ They were assembled from poly(ethylene glycol)-*b*-poly(caprolactone-*g*-trimethylene carbonate) [PEG-*b*-P(CL-*g*-TMC)] block copolymers and were therefore considered as biodegradable due to their abundant ester and carbonate bonds. We envisioned utilizing this platform to enable intracellular ring-closing metathesis—not only is this scientifically interesting but also significant as it sheds light on the broad applicability, robustness, and versatility of this platform. In this paper, we describe two routes for the integration of HGII in PEG-*b*-P(CL-*g*-TMC) polymersomes (Figure 1). HGII was either covalently bound to the block copolymer or non-covalently embedded in the polymersome membrane, making use of the hydrophobic nature of the ruthenium complex. We demonstrate that catalyst encapsulation and retention in the polymersome body limits the sudden exposure of the cell to free ruthenium, leading to lower metal-associated cellular toxicity. We further demonstrate the catalytic activity of the nanoreactors as well as their biocompatibility within living cells.

## Materials and Methods

### Materials

Organic solvents (AR or HPLC grade) were purchased from Biosolve Chemicals or Sigma-Aldrich. Methoxypoly(ethylene glycol) (1 kDa) was obtained from JenKem Technology USA. BODIPY FL carboxylic acid was acquired from Lumiprobe. Dulbecco’s modified Eagle medium (DMEM), fetal bovine serum (FBS), penicillin–streptomycin, phosphate-buffered saline (PBS), trypsin–EDTA, live cell imaging solution, cell membrane marker [wheat germ agglutinin, Alexa Fluor 594 conjugate (Alexa 594-WGA)], cell nuclear marker (Hoechst 33342), LysoTracker Red DND-99, 3-(4,5-dimethylthiazol-2-yl)-2,5-diphenyltetrazolium bromide (MTT), propidium iodide, and calcein acetoxymethyl ester (calcein AM) were purchased from ThermoFisher Scientific. All other chemicals that were used for organic synthesis were obtained from Sigma-Aldrich, Fluorochem, or TCI.

### Synthetic Procedures

The synthetic procedures of the block copolymers and the fluorogenic substrate and their corresponding characterization can be found in the Supporting Information [PEG_22_-*b*-P(CL_38_-*g*-TMC_36_), Scheme S2 and Figure S11; HGII-conjugated copolymer, Schemes S1, S3, S4 and Figures S1–S10, S12–S21; BODIPY FL-labeled copolymer, Figure S22; and fluorogenic substrate, Scheme S5 and Figures S23–S30].

### Self-Assembly of PEG-*b*-P(CL-*g*-TMC) Polymersomes by Direct Hydration

PEG_22_-*b*-P(CL_38_-*g*-TMC_36_) was dissolved in oligo(ethylene glycol) (PEG350) to make a 10 wt % solution. Optionally, a mixture of non-functionalized copolymer and BODIPY FL-labeled copolymer in a ratio of 95:5 (w/w) was used if fluorescent tracking of the polymersomes was required. Typically, 20 μL of the copolymer mixture was pipetted in a vial equipped with a magnetic stir bar and subsequently stirred to create a thin film. 80 μL of PBS (4 volume equivalents with respect to PEG350) was directly added to obtain a cloudy mixture, which was stirred for 5 min. Then, it was diluted dropwise with PBS to the desired concentration. After characterization, the polymersomes which were either non-loaded or loaded with HGII were filtered over a 0.45 μm PVDF membrane before further use.

### Self-Assembly of PEG-*b*-P(CL-*g*-TMC) Polymersomes Covalently Loaded with HGII

A 10 wt % solution of a mixture of PEG_22_-*b*-P(CL_38_-*g*-TMC_36_) and its HGII-conjugated counterpart in a ratio of 80:20 (w/w) in PEG350 was hydrated with 4 volume equivalents of PBS. After 5 min of stirring, the resulting dispersion was diluted with PBS to the desired concentration.

### Self-Assembly of PEG-*b*-P(CL-*g*-TMC) Polymersomes Non-covalently Loaded with HGII

HGII was dissolved in DMSO by 2 min of sonication under an Ar (g) atmosphere to obtain a 40 mM solution. Typically, 10 μL of a 20 wt % solution of PEG_22_-*b*-P(CL_38_-*g*-TMC_36_) in PEG350 was supplemented with 2 μL of 40 mM HGII in DMSO and 8 μL of PEG350. The resulting mixture was hydrated with 4 volume equivalents of PBS (80 μL) to induce self-assembly. Then, the dispersion was stirred for 5 min and subsequently diluted with PBS to the desired concentration.

### Ring-Closing Metathesis of the Fluorogenic Substrate Catalyzed by HGII-Loaded Polymersomes

5 μL of a 20 mM solution of the fluorogenic substrate in DMSO (100 nmol, 1.0 equiv) was diluted with 70 μL of PBS, followed by 25 μL of a 10 mg mL^–1^ dispersion of either covalently loaded or non-covalently loaded nanoreactors (7 nmol HGII, 0.07 equiv). The mixtures were incubated for 20 h under a normal air atmosphere at 37 °C. As the control, either a dispersion of plain polymersomes or free HGII catalyst (0.25 μL of a 40 mM solution in DMSO) was treated with 1 mM fluorogenic substrate in a total volume of 100 μL of PBS containing 5 vol % of DMSO. The fluorescence of the samples was measured using a microplate reader (λ_Ex_/λ_Em_ = 322 nm/440 nm), and the corresponding product concentrations were calculated using a fluorescence calibration curve of umbelliferone (Figure S41). If necessary, the samples were diluted to fall within the detection limits of the microplate reader, and this dilution was corrected during analysis. All catalytic activity assays were carried out in triplo.

### Catalytic Activity of Nanoreactors after GSH Treatment

A 20 mM GSH solution was prepared in a sodium phosphate buffer (200 mM, pH 7.4), which had been purged with Ar (g) for 0.5 h. To 90 μL of a 10 mg mL^–1^ nanoreactor dispersion (either covalently or non-covalently loaded with HGII) was added either 90 μL of a 200 mM sodium phosphate buffer or 90 μL of a 20 mM glutathione (GSH) solution in 200 mM sodium phosphate buffer. The samples were shaken at 37 °C under an Ar (g) atmosphere for 16 h, after which they were briefly cooled on ice. The polymersomes were pelleted by centrifugation (3500 rcf, 20 min, 4 °C). The supernatant was removed, and the pellets were resuspended in 90 μL of cold PBS. The polymersomes were pelleted once more and taken up in 90 μL of PBS. The remaining catalytic activity was screened by taking 25 μL of polymersome dispersion to which was added 70 μL of PBS and 5 μL of 40 mM fluorogenic substrate (in triplo). As a negative control, 2 mM fluorogenic substrate was not treated with nanoreactors. The samples were incubated at 37 °C for 20 h, after which the fluorescence was measured using a microplate reader (λ_Ex_/λ_Em_ = 322 nm/440 nm).

### Cell Viability Assay (MTT)

The toxicity of polymersomes was evaluated using an MTT cell viability assay. In accordance with the standard protocol of the MTT assay, HeLa cells (2 × 10^3^ cells/well) were seeded in 96-well tissue culture plates and cultured for 24 h. Then, polymersomes (0.1–1 mg mL^–1^ in medium/PBS 9:1) were added to the cells for 24 h of incubation. After washing the cells with PBS three times, MTT reagent was added and the cells were incubated at 37 °C for 4 h. Thereafter, MTT reagent was removed, and DMSO was added to fully dissolve the formed formazan crystals. The absorbance at 490 nm of each well was then measured using a microplate reader.

### Cell Viability Staining

HeLa cells (1 × 10^4^ cells/well) were cultured in a μ-Slide 8-well microscopy slide (glass bottom, ibidi GmbH). Thereafter, either polymersome dispersion (1 mg mL^–1^ in 200 μL of FBS-supplemented medium/PBS 9:1) or free HGII (5.6 nmol in 200 μL of FBS-supplemented medium with 0.5% DMSO) was added. After 16 h of incubation, the medium was removed and replaced with a solution of 0.1 μM calcein AM and 1 μM propidium iodide in live cell imaging solution supplemented with 0.01% DMSO. The resulting fluorescent cells were then imaged using confocal laser scanning microscopy (CLSM). This assay was also carried out using BODIPY FL-labeled polymersomes instead of non-labeled polymersomes, and calcein AM was omitted and replaced with Hoechst 33342 for nuclear staining (Figure S45).

### Cellular Colocalization Study

HeLa cells (1 × 10^4^ cells/well) were cultured in a μ-Slide 8-well microscopy slide (glass bottom, ibidi GmbH). The next day, the cells were washed with PBS. Thereafter, BODIPY FL-labeled polymersomes (1 mg mL^–1^ in FBS-supplemented medium/PBS 9:1) were added. After 16 h of incubation, the cells were washed three times with PBS. Then, the cells were stained with a nuclear marker (Hoechst 33342) for 10 min and with a colocalization marker for lysosomes (LysoTracker Red DND-99) for 30 min, followed by washings with PBS and addition of live cell imaging solution. Images were obtained by using CLSM, and colocalization of the fluorescence was analyzed with ImageJ.

### Catalytic Activity of Nanoreactors in HeLa Cells

HeLa cells (1 × 10^4^ cells/well) were cultured in a μ-Slide 8-well microscopy slide (glass bottom, ibidi GmbH). The next day, BODIPY FL-labeled polymersomes, which were covalently loaded with HGII, non-covalently loaded, or non-loaded (1 mg mL^–1^ in FBS-supplemented DMEM/PBS 9:1) were added, and the cells were incubated for 16 h. Then, the cells were washed three times with PBS, followed by the addition of 10 mM fluorogenic substrate in medium/DMSO 95:5. After 3 h of incubation at 37 °C, the HeLa cells were washed with PBS and live cell imaging solution was added. The cells were then stained with Alexa WGA-594 for 10 min. CLSM was used for imaging of the cells.

## Results and Discussion

**Scheme 1:**
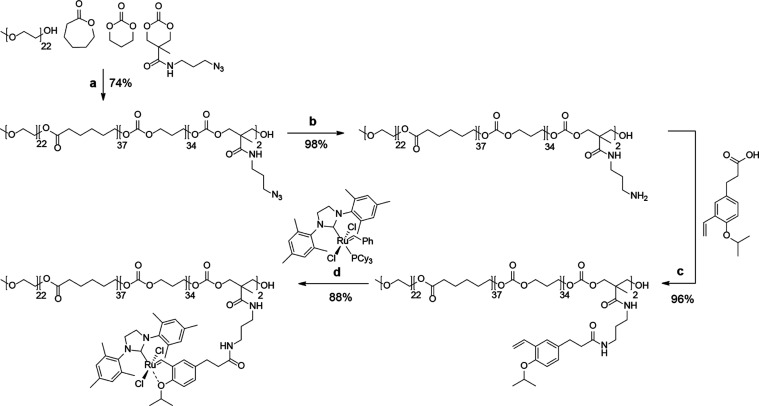
Synthesis of PEG-*g*-P(CL-*g*-TMC) Functionalized with the Hoveyda–Grubbs Second-Generation Catalyst (a) Methanesulfonic acid (MSA), CH_2_Cl_2_, rt, o/n; (b) PPh_3_, THF/H_2_O 5:1, rt, o/n; (c) 1-(*p*-isopropoxy-*m*-vinylphenyl)propionic acid, DCC, DMAP, MeCN/CH_2_Cl_2_ 5:1, rt, 2 h; (d) Grubbs Second Gen, CuCl, CH_2_Cl_2_, 35 °C, 1 h.

Utilizing our previously reported polymerization procedures, PEG_22_-*b*-P(CL_38_-*g*-TMC_36_) was synthesized via cationic ring-opening polymerization, using mPEG_22_-OH as the macroinitiator, to form the hydrophobic block as a gradient of caprolactone and trimethylene carbonate (TMC).^22−24^ To conjugate a catalyst to the hydrophobic polymer domain, a reactive monomer was co-polymerized, namely, TMC-N_3_, a TMC-analogue having a side chain functionalized with an azide (Scheme 1). Analysis of the degree of polymerization through ^1^H NMR spectroscopy showed that on average, two azides were incorporated, as demonstrated by the multiplet at δ = 3.33–3.42 ppm corresponding to two methylenes in the TMC-N_3_ side chain (Figure S17). Furthermore, the dispersity of the copolymer (*Đ* = 1.18) was determined via gel permeation chromatography (GPC) analysis (Figure S35). Post-polymerization, the azide moieties were converted to amines via a Staudinger reduction to obtain an amino-containing polymer, which was subsequently conjugated to a carboxy-functionalized styrenyl ether ligand for the HGII catalyst via an amide coupling. Finally, the polymer was equipped with ruthenium by exchanging the tricyclohexylphosphine of the Grubbs second-generation catalyst for the polymer-bound styrenyl ether. Successful complexation was confirmed by ^1^H NMR analysis, indicated by the characteristic carbene singlet at δ = 16.51 ppm (Figure S20). Moreover, we demonstrated the catalytic activity of the polymer-bound free HGII in solution by allowing it to catalyze the conversion of the model substrate *N*-tosyl diallylamine into *N*-tosyl-2,5-1*H*-dihydropyrrole in dichloromethane (Scheme S8 and Figure S34).

**Figure 2:**
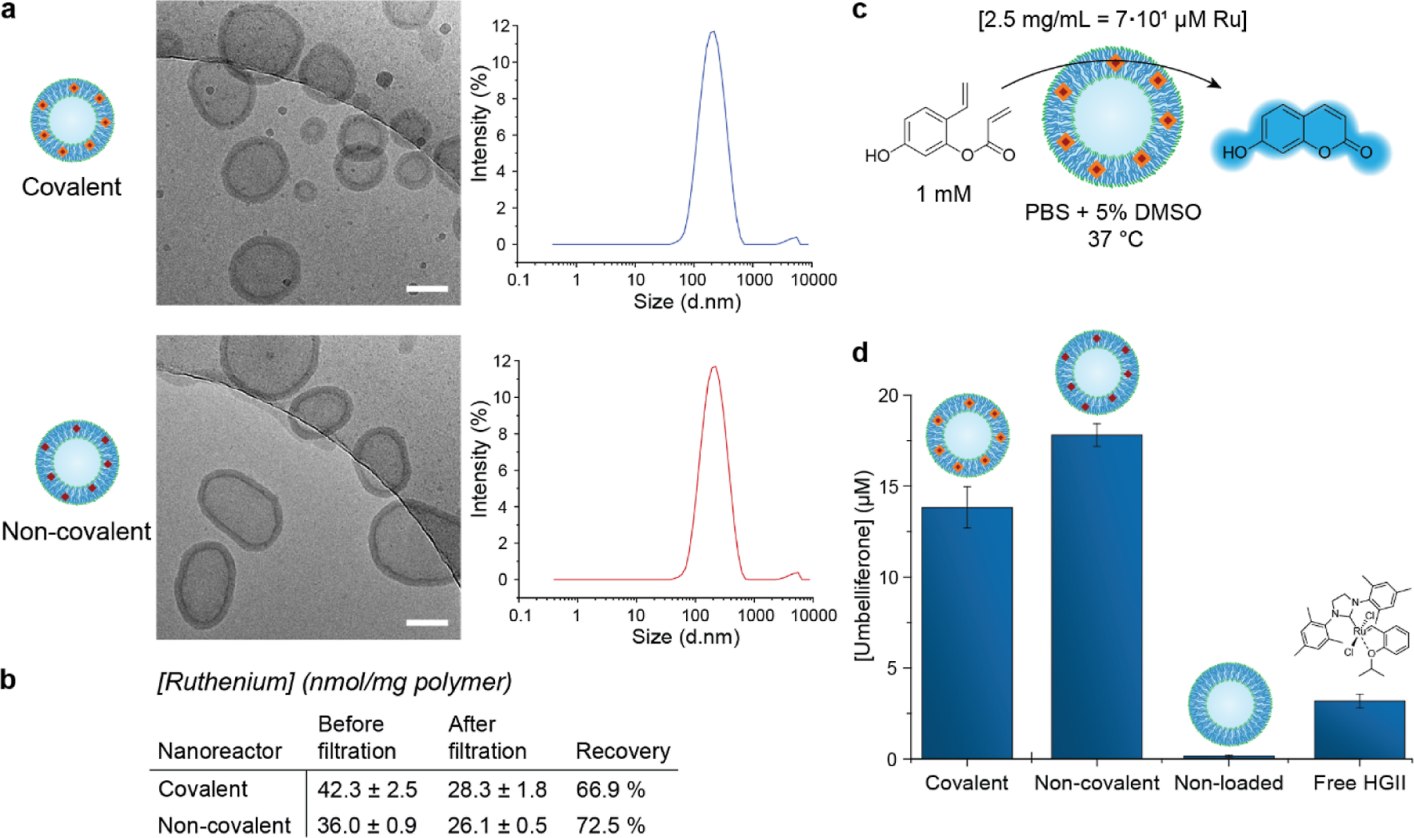
Characterization and catalytic activity of HGII-loaded nanoreactors. (a) Cryo-TEM and DLS analysis (*n* = 3) of both covalently and non-covalently loaded PEG-P(CL-*g*-TMC) polymersomes. Scale bar = 100 nm. (b) Ruthenium content of both types of nanoreactors and the recovery after filtration over a 0.45 μm membrane as determined by ICP–MS analysis. (c) Schematic representation of the conversion of the fluorogenic substrate to umbelliferone, catalyzed by the nanoreactor. (d) Nanoreactor performance based on the generation of fluorescence due to the ring-closing metathesis of the fluorogenic substrate.

Having synthesized and characterized the copolymers, we set out to test their assembly into polymersomes. Two polymersome populations were formed; one sample was prepared in which the polymersomes were functionalized with a covalently bound HGII catalyst and the other sample contained polymersomes with non-covalently incorporated HGII (Figure 1a,b, respectively). Polymersomes with covalently bound HGII were prepared by co-assembly of PEG_22_-*b*-P(CL_38_-*g*-TMC_36_) with 20–50 wt % of its HGII-functionalized counterpart. To this end, mixtures of the two copolymers were dissolved in oligo(ethylene glycol) (PEG350) and hydrated to induce self-assembly (Figure 1a). Then, their size and polydispersity (PDI) were characterized by dynamic light scattering (DLS). A copolymer ratio of 80:20 (w/w, non-functionalized copolymer/HGII-conjugated copolymer) led to the formation of polymersomes with an average size of 189 nm and a PDI of 0.21. Higher HGII–polymer contents (30–50 wt %) led to increased aggregation and a higher PDI, as observed by DLS analysis (Figure S39). Polymersomes with non-covalently incorporated HGII catalyst were formed by hydration of a combination of free HGII in DMSO and copolymer solution in PEG350 (Figure 1b). DLS analysis showed an average size of 204 nm with a PDI of 0.20. Because HGII is insoluble in water, it was assumed that the catalyst was associated with the polymersome membrane. Cryo-TEM revealed that both formulations, polymersomes with 20 wt % HGII-bound polymer and polymersomes that were non-covalently loaded with catalyst, led to the formation of spherical polymersomes (Figures 2a and S40). Both types of nanoreactors were similar in size to their non-loaded counterpart (Figure S38), demonstrating that, up to a certain wt %, the HGII catalyst did not compromise the self-assembly. These two methods of catalyst loading are different compared to the previously reported preparation of catalytic PEG-*b*-P(CL-*g*-TMC) polymersomes. Instead of loading the catalyst post-assembly, HGII was encapsulated during self-assembly, emphasizing the versatility of this platform.

The catalyst concentration of both types of nanoreactors was determined by inductively coupled plasma mass spectrometry (ICP–MS). Data analysis revealed that 1.0 mg of covalently functionalized polymersomes contained 42.3 ± 2.5 nmol of HGII (Figure 2b and Table S1), where approximately 40 nmol/mg was expected (based on the degree of functionalization of the block copolymer). The non-covalently loaded polymersomes contained 36.0 ± 0.9 nmol/mg polymer. Adequate recovery was achieved after filtration over a 0.45 μm membrane for both covalently and non-covalently loaded polymersomes, being 66.9 and 72.5%, respectively. Next, the catalytic activity of the nanoreactors was examined using a fluorogenic substrate that could be converted into blue fluorescent umbelliferone upon ring-closing metathesis (Figure 2c).^9^ Both types of nanoreactors, with covalently bound and non-covalently associated HGII, were able to promote the formation of umbelliferone, 13.8 ± 1.1 and 17.8 ± 0.6 μM, respectively (Figure 2d). These conversions were higher compared to the formation of 3.2 ± 0.4 μM of umbelliferone as catalyzed by free HGII, likely due to its lack of solubility in aqueous solutions. The “non-covalent” nanoreactors resulted in a slightly higher conversion compared to their covalent analogues. This could be attributed to the free diffusion of the catalyst within the polymersomes, leading to better accessibility for the substrate. As expected, treatment of the substrate with non-loaded polymersomes did not generate any fluorescence. Although the nanoreactors performed better than free HGII under these conditions, conversions were still rather low (in comparison to the initial substrate concentration of 1.0 mM). Such low conversions can be attributed to (1) the electron-withdrawing effect of the ester in the acrylate, which significantly hampers the progression of ring-closing metathesis^25^ and, more importantly, (2) the protective polymersome membrane, which forms a diffusion barrier for the substrate.

**Figure 3:**
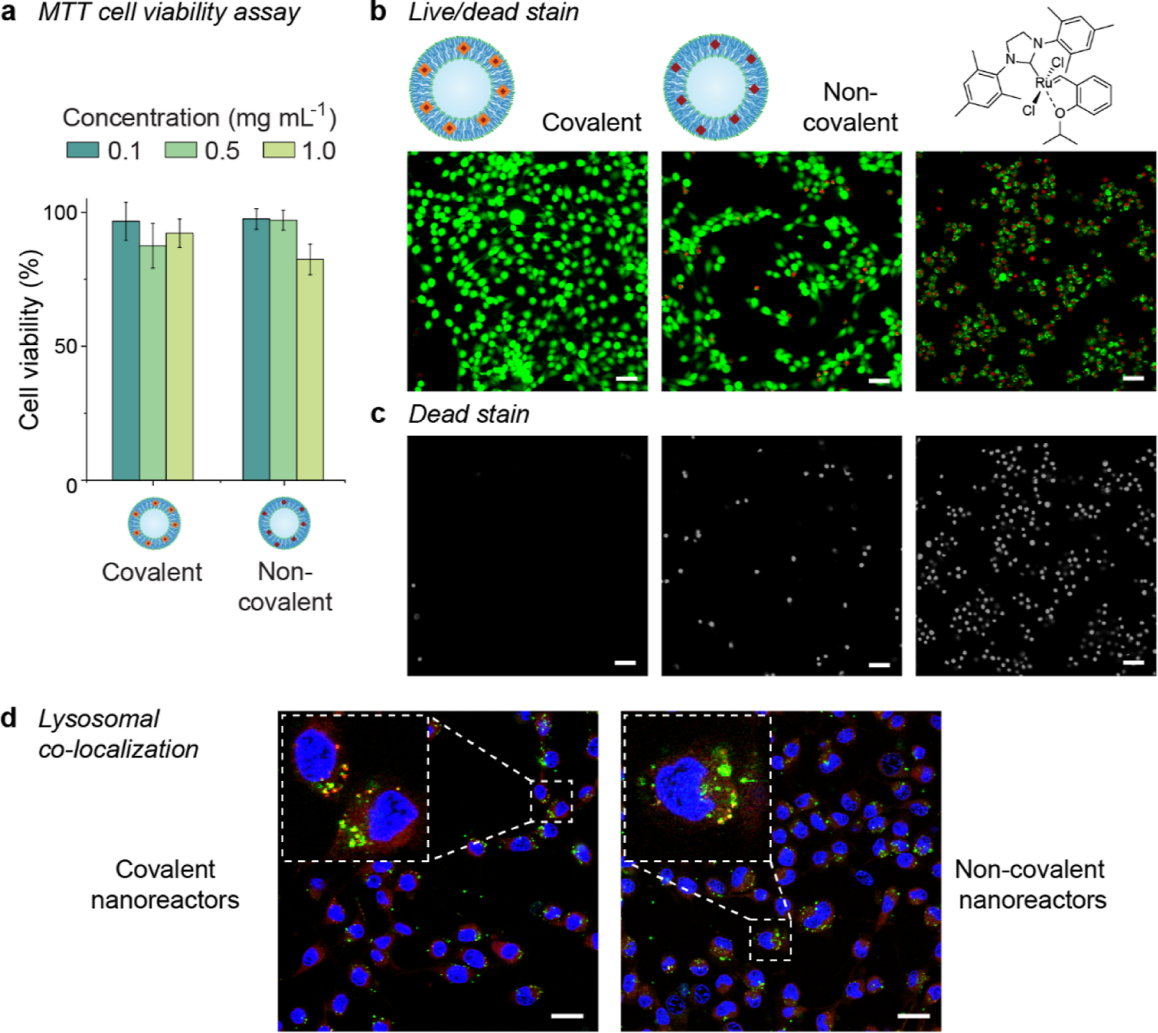
Cytotoxicity and localization of covalently and non-covalently HGII-loaded nanoreactors in HeLa cells after 16 h of treatment. (a) Cell viability determined by the MTT assay. (b) Live/dead staining with calcein AM and PI of cells treated with 1 mg mL^–1^ of nanoreactor or 5.6 nmol of free HGII. (c) Dead stain only, in grayscale. (d) Colocalization of BODIPY FL-labeled nanoreactors (green) and LysoTracker Red (red) in HeLa cells of which the nuclei were stained with Hoechst. Overlap of the green and red signals results in yellow, indicating colocalization. Scale bar = 50 μm in (b,c) and 25 μm in (d).

Crucial to the application of our nanoreactors as artificial organelles in living cells is their biocompatibility. Therefore, we investigated their cytotoxicity using a range of different concentrations of polymersomes via the MTT assay (Figure 3a). Up to 1.0 mg mL^–1^, both covalently bound and non-covalently HGII-loaded polymersomes barely affected the viability of HeLa cells. Additionally, this was investigated by CLSM using a live/dead stain involving calcein AM and propidium iodide (PI) (Figure 3b,c). In parallel with incubation with nanoreactors (1.0 mg mL^–1^), cells were incubated with free HGII, keeping the amount of ruthenium nearly equal for all conditions. Almost all of the cells that were treated with the “covalent” nanoreactors remained viable. The non-covalent nanoreactors were slightly more toxic according to this method; however, most cells still showed good viability. In contrast, all cells treated with free HGII showed red fluorescence, indicating cell death (Figure 3b,c). These findings confirm the need for encapsulation of this type of catalyst. The cellular fate of the polymersomes is of interest to help understand where in the cell the catalytic conversion of a potential substrate takes place. Fluorescently labeled nanoreactors of both types were found to co-localize with lysosomes, as demonstrated by staining with LysoTracker Red (Pearson correlation coefficient *r* = 0.40 with Costes’ statistical significance *P* = 1.00 for both nanoreactor types) (Figure 3d).

**Figure 4:**
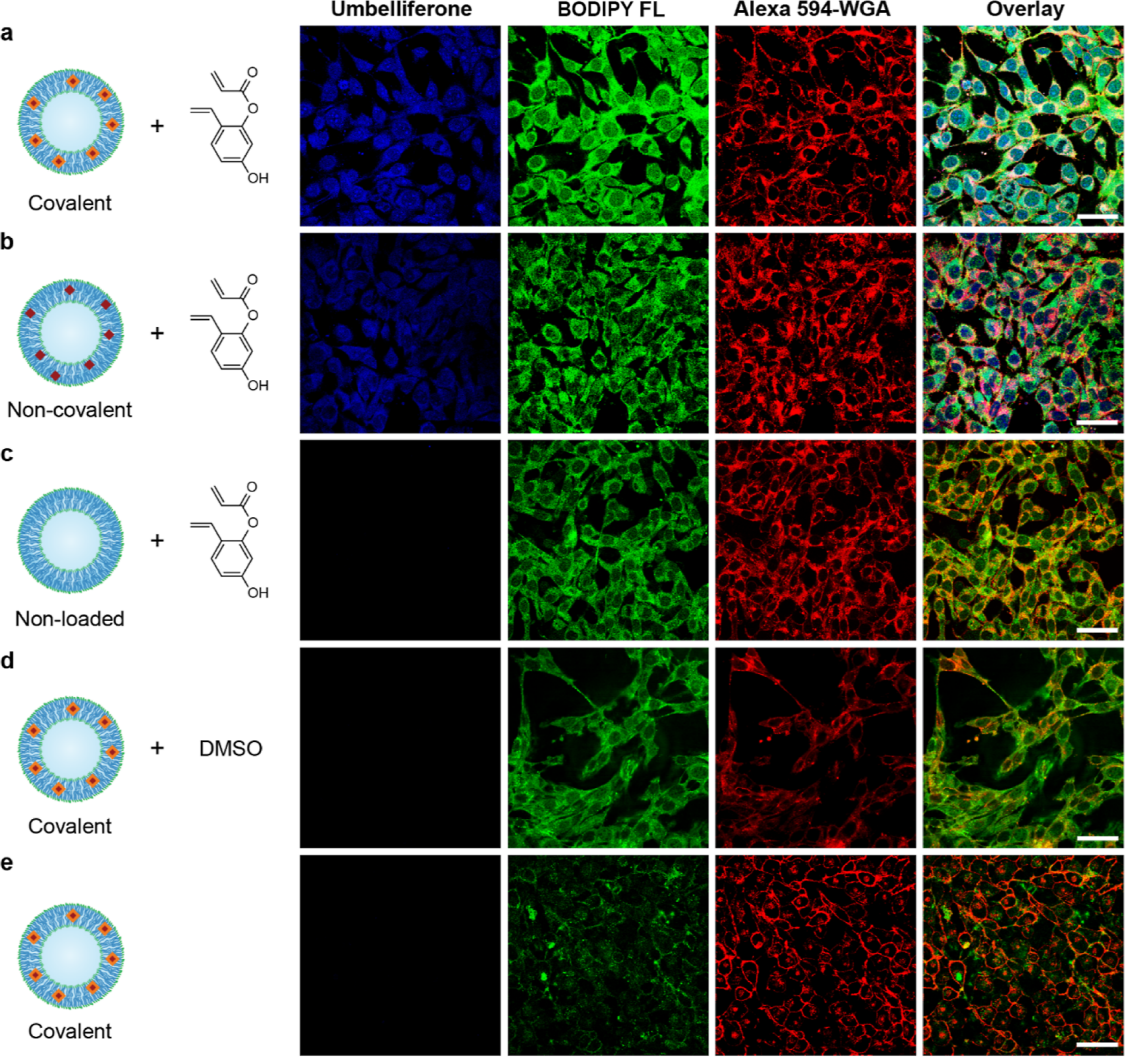
HGII nanoreactors as artificial organelles. HeLa cells were incubated with covalently loaded (a,d), non-covalently loaded (b), or non-loaded (c) polymersomes for 16 h. Thereafter, they were washed and treated with 10 mM substrate in medium/DMSO 95:5 (a–c), medium/DMSO 95:5 without substrate (d), or medium only (e). Besides product formation, increased green fluorescence was observed, which was attributed to autofluorescence as a result of DMSO-associated oxidative stress.^30−34^ Indeed, cells treated with only nanoreactors did not show this increased autofluorescence (e). Scale bar = 50 μm.

Finally, we investigated the activity of our two types of artificial organelles. HeLa cells were incubated with BODIPY FL-labeled catalytic vesicles (1 mg mL^–1^) to allow for tracking of cellular uptake. Hereafter, the cells were washed and incubated with 10 mM fluorogenic substrate in 5 vol % DMSO for 3 h. This was followed by cell membrane staining and imaging by CLSM. Unlike the control cells, which were treated with non-functionalized polymersomes, the cells became blue fluorescent due to the intracellular generation of umbelliferone by either type of nanoreactor via HGII-catalyzed ring-closing metathesis, regardless of the hostile intracellular environment (Figure 4a–c). Since GSH is able to deactivate the catalyst,^9^ we were interested to find out if catalyst deprotection is a result of hindered diffusion of GSH across the polymersome membrane or a result of the specific location of the polymersomes within the cell. To address this, glutathione was added to both types of nanoreactors without cells present. Then, glutathione was removed again and the nanoreactor activity was monitored. Indeed, despite GSH’s charged nature, exposure of nanoreactors to a GSH concentration of 10 mM led to their deactivation (Figure S42). Therefore, the most plausible explanation for the intracellular activity lies in the localization of the artificial organelles. The polymersomes direct the catalyst to endosomal compartments upon cellular uptake. Even though it is still under debate, evidence suggests that these local environments are relatively oxidizing, meaning more GSH is present in its dimeric GSSG state, compared to that present in the cytosol.^26−29^ Evidently, the GSH concentration in the endosomes is low enough for the nanoreactors to remain active; otherwise, the cells would not have become blue fluorescent.

## Conclusions

We have developed biodegradable polymersome nanoreactors with either covalently linked or non-covalently embedded HGII catalysts. Whereas the free catalyst was highly toxic, the nanoreactors showed good biocompatibility by retaining the catalyst and thereby preventing exposure to high amounts of free catalyst. Inside the endo/lysosomes of HeLa cells, the nanoreactors were able to convert a fluorogenic substrate via ring-closing metathesis, resulting in blue fluorescent cells. Crucial to this intracellular catalytic activity was the endo/lysosomal localization inside the cells. The polymersome membrane was shown not to offer enough protection against glutathione, and therefore, they need an environment with a lower GSH concentration to remain catalytically active, such as the endosome. Interestingly, both the covalent and non-covalent approaches worked equally well, which indicates that in the former case, the catalytic moiety is tolerated during polymersome assembly and that in the latter case, catalyst leaching is not an issue. This is the first example of the use of a polymersome-based artificial organelle with an active ruthenium catalyst for carbon–carbon bond formation. It would be interesting to expand this platform by having more than one type of catalyst per nanoreactor, which could be located in the membrane, and the aqueous lumen of the polymersome.

## Supporting Information Available

Synthetic procedures of the copolymers and the corresponding NMR, IR, and GPC analysis, synthetic procedures and characterization of the fluorogenic substrate, DLS and cryo-TEM analysis of nanoreactors, fluorescence calibration curve of umbelliferone, and catalytic activity of nanoreactors after GSH treatment (PDF)
