# Supplementary material for: Biodegradable Grubbs-Loaded Artificial Organelles for Endosomal Ring-Closing Metathesis
Source: Biomacromolecules. 2023 Aug 17;24(9):4148–55. doi: 10.1021/acs.biomac.3c00487 (PMC10498438; doi:10.1021/acs.biomac.3c00487)
Supplement: Supplementary file 1 — bm3c00487_si_001.pdf [file bm3c00487_si_001.pdf]

Supporting information

Biodegradable Grubbs-Loaded Artificial Organelles for Endosomal Ring-Closing Metathesis

Roy A. J. F. Oerlemans, Jingxin Shao, Marleen H. M. E. van Stevendaal, Hanglong Wu, Tania Patiño Padial, Loai K. E. A. Abdelmohsen, Jan C. M. van Hest**

1. Instrumentation

Nuclear Magnetic Resonance Spectroscopy

Nuclear magnetic resonance spectra (^{13}C NMR, ^{1}H NMR and ^{19}F NMR) were recorded on a 400 MHz Bruker Cryomagnet or 400 MHz Varian Gemini. Chemical shifts (in ppm) were referenced to the residual non-deuterated solvent, relative to tetramethylsilane (TMS) as internal standard.

Liquid Chromatography-Mass Spectrometry

The liquid chromatography-mass spectrometry (LC-MS) system used, was equipped with a Phenomex kinetex 2.6 µm EVO C18 50x2.1 mm column and run with a gradient of MeCN (5-100%, with 0.1% formic acid) in ultrapure water (with 0.1% formic acid) over 10 minutes, monitored by a PDA detector and a Thermo Scientific LCQ Fleet Ion Trap Mass Spectrometer.

Gas Chromatography-Mass Spectrometry

For gas chromatography-mass spectrometry (GC-MS) analysis, a Phenomenex Zebron ZB-5MS 30 m x 0.25 mm x 0.25 mm column was used with a gradient of 80 °C - 300 °C (30 °C per minute, in helium gas), coupled to a GC-MS-QP2010 Plus Quadrupole Mass Spectrometer.

Matrix-assisted laser desorption/ionisation-time of flight mass spectrometry

stainingMatrix-assisted laser desorption/ionisation-time of flight mass spectrometry (MALDI-TOF MS) spectra were recorded on a PerSeptive Biosystems Voyager-DE Pro spectrometer using either of the following matrices: α-cyano-4-hydroxycinnamic acid (CHCA) or 2-[(2E)-3-(4-*tert*-butylphenyl)-2-methylprop-2-enylidene]malononitrile (DCTB).

Infrared Spectroscopy

Infrared spectra were obtained with a PerkinElmer FT-IR Spectrum Two.

Gel Permeation Chromatography

Gel permeation chromatography (GPC) was performed on a Prominence-I GPC system (Shimadzu) with PL gel 5 µm mixed D column (Polymer Laboratories), eluted with THF at a rate of 1.0 mL min^{-1} and equipped with a RID-20A differential refractive index detector and a PDA detector.

GPC with DMF supplemented with 10 mM LiBr as eluent (1.0 mL min^{-1}) was performed using a KD-804 column (Shodex).

Dynamic Light Scattering

Dynamic light scattering (DLS) was measured on a Malvern Zetasizer Nano ZSP equipped with 633 nm laser.

Inductively Coupled Plasma-Mass Spectrometry

Inductively coupled plasma-mass spectrometry (ICP-MS) analysis was performed on a Thermo Scientific Xseries I quadrupole using $InCl_3$ as internal standard.

Cryogenic Transmission Electron Microscopy

Cryogenic transmission electron microscopy (cryo-TEM) was imaged using a CryoTitan (ThermoFisher Scientific). The machine was equipped with a field-emission gun which was operating at 300 kV, as well as a post-column Gatan energy filter and an autoloader station. The grids (R 2/2, Cu, Quantifoil Jena grids, Quantifoil Micro Tools GmbH) were first plasma-treated in a Cressington 208 carbon coater for 40 seconds. Then, 3.0 µL of sample solution was pipetted onto the grid and blotted in a Vitrobot MARK IV (ThermoFisher Scientific) at room temperature with 100% humidity. The grid was blotted for 3.0 seconds (blotting force -3) and directly plunged and frozen in liquid ethane. The images were acquired using a 2k Gatan CCD (charged-coupled device) camera (model 794). Hereafter, the images were analyzed using ImageJ, provided for free by the National Institute of Health, USA.

Fluorescence Spectroscopy

Fluorescence measurements were performed on a TECAN Spark 10M microplate reader using Nunc MicroWell 96-well black-bottom polystyrene plates (ThermoFisher Scientific).

Cell culture

Human cervical carcinoma (HeLa) cells were grown in DMEM supplemented with 10% (v/v) FBS and 1% (v/v) penicillin-streptomycin in an incubator (ThermoFisher Scientific) under a humidified atmosphere at 37 °C with 5% CO_2.

Confocal Laser Scanning Microscopy

Fluorescence in live cells was imaged by confocal laser scanning microscopy using a Leica TCS SP8X equipped with microscope incubator (Okolab) to keep the HeLa cells at 37 °C and 5% CO_2 during observation. Images were acquired with a resolution of 1024×1024 pixels and a scan rate of 400 Hz by a 63×oil immersion objective.

Column chromatography

Column chromatography was performed either manually (silica gel, 40-63 µm particle size) or automated by using a Grace Reveleris X2 or Biotage Isolera with prepacked silica gel columns (40 µm particle size) (Büchi or Grace).

2. Experimental procedures

All reactions were carried out under a protective atmosphere of Ar (g) unless stated otherwise.

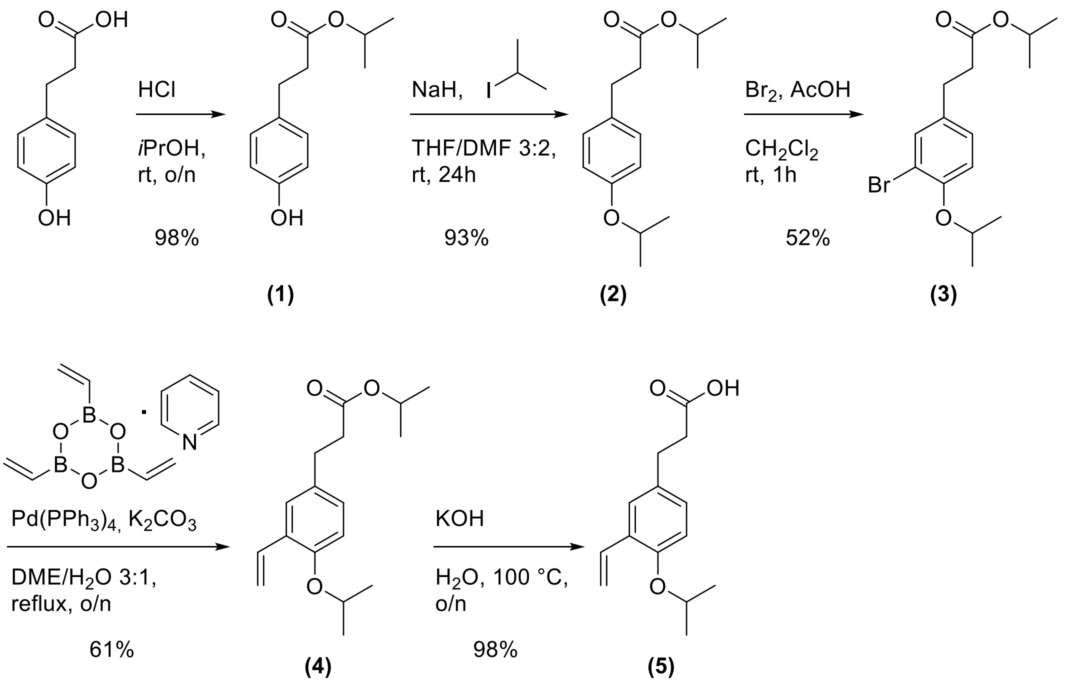

Scheme S1. *Synthesis of 1-(p-isopropoxy-m-vinylphenyl)propionic acid (**5**).*

Isopropyl-1-(*p*-hydroxyphenyl)propionate (1) was synthesized according to literature procedure with modifications.[1]

3-(4-hydroxyphenyl)propionic acid (8.00 g, 48.1 mmol) was dissolved in 100 mL 6M HCl in isopropanol. The solution was stirred overnight at room temperature under Ar (g) atmosphere. The mixture was added dropwise to a solution of 84 mL triethylamine in 2-propanol at 0 °C. The resulting mixture was concentrated under reduced pressure and the residue was taken up in CH_2Cl_2. This was washed once

with 0.1 mM $NaHCO_3$ (aq) and twice with deionised water. The organic phase was dried over $MgSO_4$ and concentrated under reduced pressure to give **1** as an off-white powder (9.83 g, 47.2 mmol, 98%).

^{1}H NMR (400 MHz, Chloroform-*d*) δ = 7.11 – 6.98 (m, 2H), 6.80 – 6.68 (m, 2H), 5.70 (s, 1H), 5.00 (hept, *J*=6.3, 1H), 2.86 (t, *J*=7.7, 2H), 2.56 (t, *J*=7.7, 2H), 1.20 (d, *J*=6.3, 6H).
^{13}C NMR (101 MHz, Chloroform-*d*) δ = 173.10, 154.21, 132.38, 129.41, 115.31, 68.00, 36.62, 30.21, 21.80.
GC-EI MS [M] $^{\bullet+}$ calcd. 208 Da, found 208 Da.

Isopropyl-1-(*p*-isopropoxyphenyl)propionate (2) was synthesized according to literature procedure with modifications.[1]

A slurry of 60% sodium hydride in mineral oil (1.06 g, 26.4 mmol, 1.10 eq.) was washed twice with 3 mL pentane under Ar (g) atmosphere and suspended in 50 mL anhydrous THF. This was added dropwise to a solution of compound **1** (5.00 g, 24.0 mmol, 1.0 eq.) in 50 mL of anhydrous THF at 0 °C under Ar (g) atmosphere. Next, a solution of 2-iodopropane (2.50 mL, 25.2 mmol, 1.05 eq.) in 70 mL anhydrous DMF was added dropwise. The mixture was stirred overnight at room temperature under Ar (g) atmosphere. Additional sodium hydride and 2-iodopropane were added until full conversion was observed by TLC analysis (silica gel, heptane/EtOAc, 95:5). The reaction mixture was concentrated under reduced pressure to approximately 100 mL and subsequently diluted with 500 mL CH_2Cl_2. This was washed 3 times with brine, 3 times with deionised water and once more with brine, after which the organic phase was dried over $MgSO_4$ and concentrated under reduced pressure. The resulting red oil was purified by flash chromatography (heptane/ethyl acetate gradient 95:5-90:10) to obtain **2** as a colorless oil (5.59 g, 22.3 mmol, 93%).

^{1}H NMR (400 MHz, Chloroform-*d*) δ = 7.14 – 7.05 (m, 2H), 6.85 – 6.77 (m, 2H), 4.99 (hept, *J*=6.3, 1H), 4.50 (d, *J*=6.1, 1H), 2.87 (t, *J*=7.8, 2H), 2.55 (t, *J*=7.8, 2H), 1.31 (d, *J*=6.1, 6H), 1.20 (d, *J*=6.3, 6H).
^{13}C NMR (100 MHz, Chloroform-*d*) δ = 172.55, 156.30, 132.55, 129.25, 115.93, 69.90, 67.60, 36.54, 30.22, 22.08, 21.82.
GC-EI-MS [M] $^{\bullet+}$ calcd. 250 Da, found 250 Da.

Isopropyl-1-(*m*-bromo-*p*-isopropoxyphenyl)propionate (3) was synthesized according to literature procedure with modifications.[1]

To a solution of **2** (4.50 g, 18.0 mmol, 1.0 eq.) in 70 mL of anhydrous CH_2Cl_2, glacial acetic acid (41 µL, 0.7 mmol, 0.04 eq.) was added under Ar (g) atmosphere. A solution of bromine (0.930 mL, 18.2 mmol, 1.01 eq.) in 20 mL anhydrous CH_2Cl_2 was added dropwise and the reaction mixture was stirred for 1 hour at room temperature under Ar (g) atmosphere, after which the reaction was quenched with 20 mL saturated sodium thiosulfate. The mixture was diluted with 100 mL CH_2Cl_2 and washed once with deionised water and once with brine. The organic phase was dried over $MgSO_4$ and concentrated under reduced pressure. The resulting yellow oil was purified by flash chromatography (silica gel, heptane/Et_2O gradient 97:3-90:10) to give 3.83 g of a colorless oil containing product as well as traces of starting material and dibrominated adduct. Additional flash chromatography (CH_2Cl_2/pentane 3:2) yielded **3** as a colorless oil (3.10 g, 9.42 mmol, 52%).

^{1}H NMR (400 MHz, Chloroform-*d*) δ = 7.38 (d, *J*=2.2, 1H), 7.06 (dd, *J*=8.4, 2.2, 1H), 6.83 (d, *J*=8.4, 1H), 5.00 (hept, *J*=6.3, 1H), 4.49 (hept, *J*=6.1, 1H), 2.85 (t, *J*=7.7, 2H), 2.55 (t, *J*=7.7, 2H), 1.36 (d, *J*=6.1, 6H), 1.20 (d, *J*=6.3, 6H).
^{13}C NMR (101 MHz, Chloroform-*d*) δ = 172.21, 152.99, 134.49, 133.21, 128.13, 116.09, 113.73, 72.37, 67.80, 36.24, 29.85, 22.09, 21.83.
GC-EI-MS [M] $^{\bullet+}$ calcd. 328 Da, found 328 Da.

Isopropyl-1-(*p*-isopropoxy-*m*-vinylphenyl)propionate (4)

A solution of **3** (1.50 g, 4.56 mmol, 1.0 eq.) in 60 mL of 1,2-dimethoxyethane (DME) was purged with Ar (g) for 30 minutes. To this tetrakis(triphenylphosphine)palladium (263 mg, 0.228 mmol, 0.05 eq.) was added and the solution was stirred under Ar (g) atmosphere. A solution of potassium carbonate (630 mg, 4.56 mmol, 1.0 eq.) in 18 mL MilliQ water, previously purged with Ar (g), was added to the reaction mixture, followed by the vinylboronic anhydride pyridine complex (1.10 g, 4.56 mmol, 1.0 eq.). The mixture was heated at reflux for 20 hours under Ar (g) atmosphere, after which it was allowed to cool

down to room temperature. Then it was extracted once with 120 mL CH_2Cl_2, and twice with 20 mL CH_2Cl_2. The organic layers were combined and washed with brine, dried over Na_2SO_4, filtered over celite and concentrated under reduced pressure to give a yellow oil. This was purified by flash chromatography (silica gel, heptane/Et_2O 8:1) to give **4** as a colorless oil (768 mg, 2.78 mmol, 61%).

^{1}H NMR (399 MHz, Chloroform-*d*) δ = 7.30 (d, *J*=2.2, 1H), 7.11 – 6.93 (m, 2H), 6.80 (d, *J*=8.4, 1H), 5.71 (dd, *J*=17.8, 1.6, 1H), 5.21 (dd, *J*=11.2, 1.6, 1H), 5.00 (hept, *J*=6.3, 1H), 4.48 (hept, *J*=6.0, 1H), 2.88 (t, *J*=7.8, 2H), 2.56 (t, *J*=7.8, 2H), 1.33 (d, *J*=6.0, 6H), 1.20 (d, *J*=6.3, 6H).
^{13}C NMR (100 MHz, Chloroform-*d*) δ = 172.54, 153.67, 132.66, 131.96, 128.49, 127.82, 126.33, 114.54, 113.84, 71.06, 67.65, 36.54, 30.36, 22.22, 21.84.
GC-EI-MS $[M]^{\bullet +}$ calcd. 276 Da, found 276 Da.

1-(*p*-Isopropoxy-*m*-vinylphenyl)propionic acid (5) was synthesized according to literature procedure with modifications.[1]

To 7.3 mL 1M KOH (aq) (7.30 mmol, 40 eq.), **4** (50.0 mg, 0.181 mmol, 1.0 eq.) was added. The mixture was stirred at 100 °C overnight. Then it was cooled down to 0 °C and neutralized to a pH between 7 and 8 using 1M $KHSO_4$ (aq) (as the product is prone to polymerization under acidic conditions). A white precipitate appeared, which was extracted with 15 mL Et_2O. The organic phase was dried over Na_2SO_4 to give **5** as a white solid (41.5 mg, 0.177 mmol, 98%).

^{1}H NMR (399 MHz, Chloroform-*d*) δ = 10.38 (s, 1H), 7.31 (d, *J*=2.2, 1H), 7.13 – 6.94 (m, 2H), 6.81 (d, *J*=8.4, 1H), 5.72 (dd, *J*=17.8, 1.5, 1H), 5.22 (dd, *J*=11.1, 1.5, 1H), 4.49 (hept, *J*=6.1, 1H), 2.90 (t, *J*=7.8, 2H), 2.66 (t, *J*=7.8, 2H), 1.33 (d, *J*=6.1, 6H).
^{13}C NMR (100 MHz, Chloroform-*d*) δ = 179.21, 153.79, 132.19, 131.92, 128.42, 127.92, 126.33, 114.55, 114.02, 71.06, 35.92, 29.92, 22.23.
MALDI-TOF MS $[M]^{\bullet +}$ calcd. 234.13 Da, found 234.13 Da.

PEG_{22}-*b*-P(CL_{38}-*g*-TMC_{36}) (6) was synthesized according to literature procedures.[2,3]

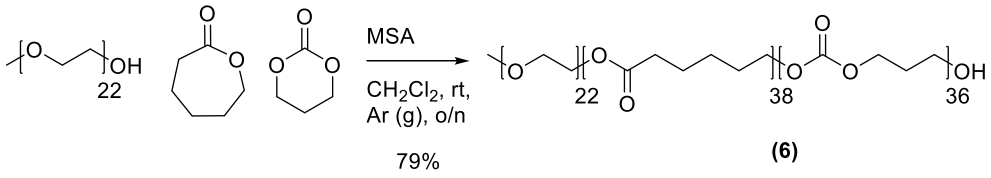

Scheme S2. *Synthesis of PEG_{22}-b-P(CL_{38}-g-TMC_{36}) (**6**).*

Poly(ethylene glycol) (Mn ~1.000 Da) (150 mg, 0.15 mmol, 1.0 eq.), previously lyophilized from dioxane, trimethylene carbonate (540 mg, 5.25 mmol, 35 eq.) and ε-caprolactone (582 µL, 5.25 mmol, 35 eq.) were treated twice with anhydrous toluene to azeotropically remove residual traces of water by concentration under reduced pressure. The residue was dissolved in 27 mL anhydrous CH_2Cl_2 under Ar (g) atmosphere. Methanesulfonic acid (30 µL, 0.45 mmol, 3.0 eq.) was added and the solution was stirred for 24 hours at room temperature under Ar (g) atmosphere. An aliquot was quenched with triethylamine and analyzed by ^{1}H NMR spectroscopy to confirm that the reaction approached completion. The reaction mixture was quenched with saturated $NaHCO_3$ (aq). The organic phase was washed with brine, dried over Na_2SO_4 and concentrated under reduced pressure. The resulting colorless oil was further purified by precipitation in cold Et_2O. Finally, it was lyophilized from dioxane to obtain **6** as a colorless wax (1.02 g, 79%, Đ= 1.17).

^{1}H NMR (400 MHz, Chloroform-*d*) δ = 4.34 – 4.00 (m), 3.64 (s), 3.38 (s), 2.39 – 2.25 (m), 2.10 – 1.95 (m), 1.74 – 1.59 (m), 1.46 – 1.33 (m).

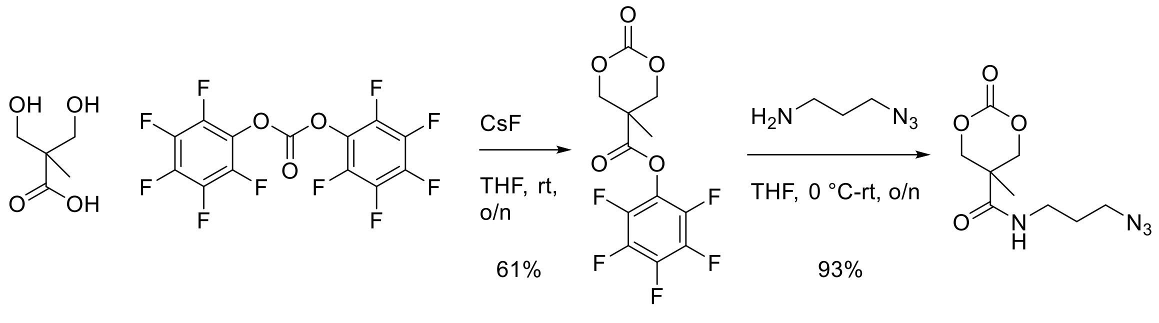

Scheme S3. *Synthesis of TMC-propyl-N_3 (**8**).*

Pentafluorophenyl 5-methyl-2-oxo-1,3-dioxane-5-carboxylate (TMC-PFP) (7) was synthesized according to literature procedure.[4]

A suspension of 2,2-bis(hydroxymethyl)propionic acid, (1.36 g, 10.1 mmol, 1.0 eq.), bis-(pentafluorophenyl)carbonate (10.0 g, 25.4 mmol, 2.5 eq.) and cesium fluoride (0.32 g, 2.1 mmol, 0.21 eq.) in anhydrous THF was stirred under Ar (g) atmosphere. After 15 minutes, the mixture turned into a solution. This was stirred for 20 hours under Ar (g) atmosphere, after which it was concentrated under reduced pressure and subsequently redissolved in CH_2Cl_2. The precipitated by-product, pentafluorophenol, was removed by filtration. The filtrate was washed twice with saturated $NaHCO_3$ (aq) and once with deionised water. The organic phase was dried over $MgSO_4$ and concentrated under reduced pressure. The residue was crystallized from EtOAc/heptane to give TMC-PFP (**7**) as a white powder (1.99 g, 61.1 mmol, 61%).

^{1}H NMR (399 MHz, Chloroform-*d*) δ = 4.85 (d, *J*=10.8, 2H), 4.37 (d, *J*=10.8, 2H), 1.55 (s, 3H).
^{13}C NMR (100 MHz, Chloroform-*d*) δ = 167.89, 146.74, 142.36 – 136.34 (m), 124.36, 72.45, 41.04, 17.49.
^{19}F NMR (376 MHz, Chloroform-*d*) δ = -152.71 – -152.88 (m, 2F), -156.11 (t, *J*=21.8, 1F), -161.08 – -161.37 (m, 2F).
MALDI-TOF MS $[M+Na]^+$ calcd. 349.01 Da, found 349.02 Da.

***N*-(3-azidopropyl)-5-methyl-2-oxo-1,3-dioxane-5-carboxamide (TMC-propyl-N_3) (8)**

A suspension of TMC-PFP (**7**) (326 mg, 1.00 mmol, 1.0 eq.) in 2 mL anhydrous THF was stirred under Ar (g) atmosphere at 0 °C. Azidopropanamine (98.2 µL, 1.00 mmol, 1.0 eq.) was added dropwise and the mixture was stirred overnight under Ar (g) atmosphere, while slowly warming to room temperature. The solution was concentrated under reduced pressure and purified by flash chromatography (silica gel, heptane/EtOAc gradient 50:50-0:100) to give TMC-propyl-N_3 (**8**) as a colorless oil (225 mg, 0.93 mmol, 93%).

^{1}H NMR (400 MHz, Chloroform-*d*) δ = 6.91 (t, *J*=5.8, 1H), 4.72 (d, *J*=10.8, 2H), 4.25 (d, *J*=10.8, 2H), 3.46 – 3.31 (m, 4H), 1.81 (p, *J*=6.6, 2H), 1.33 (s, 3H).
^{13}C NMR (101 MHz, Chloroform-*d*) δ = 170.34, 148.19, 73.87, 49.36, 39.91, 37.76, 28.34, 17.66.
MALDI-TOF MS $[M+Na]^+$ calcd. 265.09 Da, found 265.09 Da.

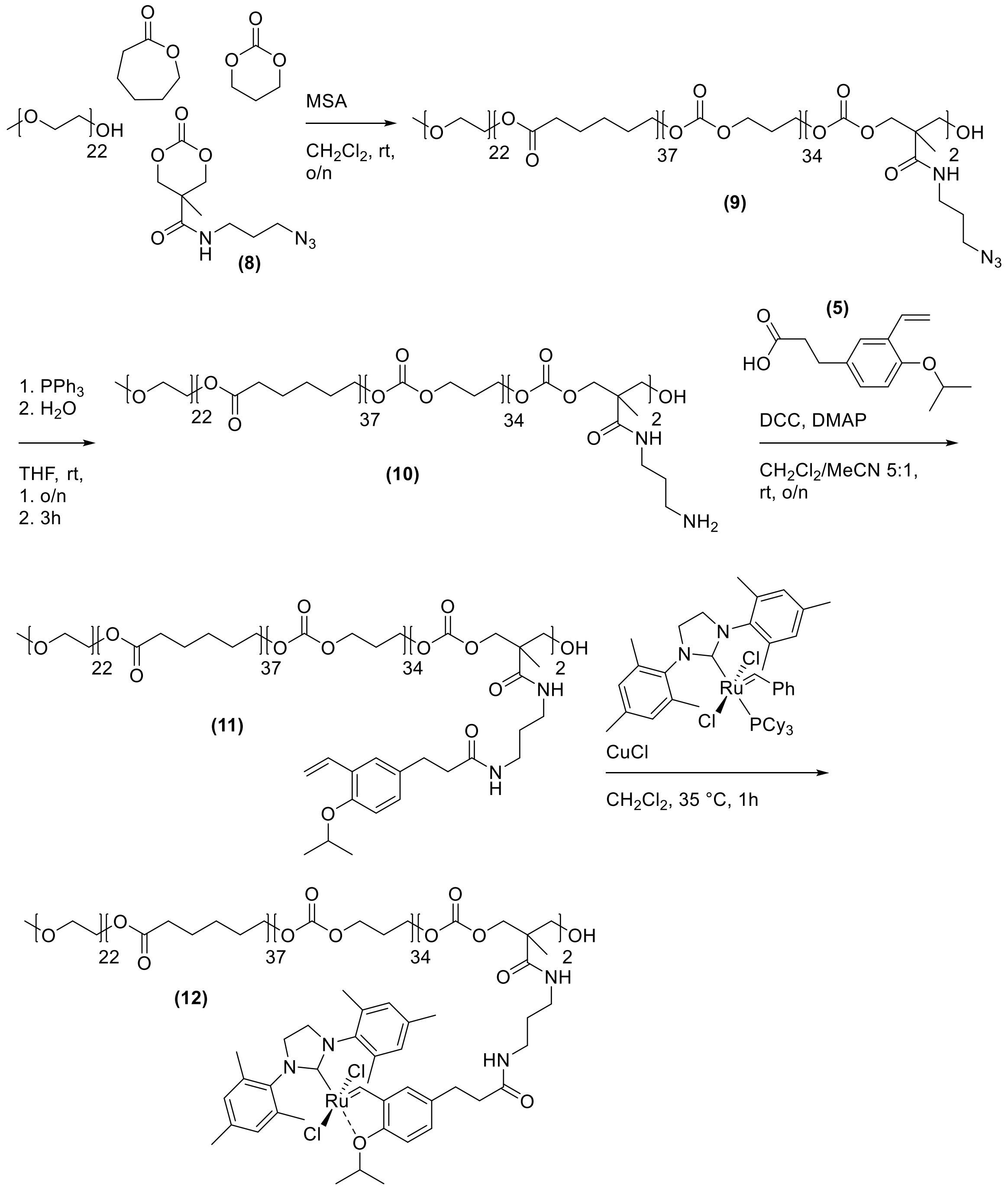

Scheme S4. *Synthesis of HGII-conjugated PEG-b-P(CL-g-TMC) (**12**).*

N_3-block copolymer PEG_{22}-*b*-P(CL_{37}-*g*-TMC_{34}-*g*-(TMC-N_3)$_2$) (9)

Poly(ethylene glycol) (Mn ~1.000 Da) (42 mg, 0.04 mmol, 1.0 eq.), previously lyophilized from dioxane, TMC-propyl-N_3 (**8**) (26 mg, 0.11 mmol, 2.56 eq.), trimethylene carbonate (143 mg, 1.39 mmol, 33 eq.) and ε-caprolactone (158 µL, 1.43 mmol, 34 eq.) were treated twice with anhydrous toluene to azeotropically remove residual traces of water by concentration under reduced pressure. The residue was dissolved in 8 mL anhydrous CH_2Cl_2 under Ar (g) atmosphere. Methanesulfonic acid (16 µL, 0.15 mmol, 6.0 eq.) was added and the solution was stirred for 24 hours at room temperature under Ar (g) atmosphere. An aliquot was quenched with triethylamine and analyzed by ^1H NMR spectroscopy to confirm that the reaction approached completion. The reaction mixture was quenched with saturated $NaHCO_3$ (aq). The organic phase was washed with brine, dried over Na_2SO_4 and concentrated under reduced pressure. The resulting colorless oil was further purified by precipitation in cold Et_2O. Finally, it

was lyophilized from dioxane to obtain N_3-block copolymer (**9**) as a colorless wax (276 mg, 74%, Đ= 1.18). IR spectroscopy analysis showed the characteristic azide signal at 2100 cm^{-1} (Fig. S37).

^{1}H NMR (400 MHz, Chloroform-*d*) δ = 4.43 – 3.99 (m), 3.65 (s), 3.42 – 3.33 (m), 2.41 – 2.23 (m), 2.11 – 1.87 (m), 1.83 – 1.77 (m), 1.75 – 1.56 (m), 1.47 – 1.32 (m), 1.30 – 1.15 (m).

NH_2-block copolymer PEG_{22}-*b*-P(CL_{37}-*g*-TMC_{34}-*g*-(TMC-NH_2)$_2$) (10)

Anhydrous THF was purged with Ar (g) for 15 minutes. N_3-block copolymer (**9**) (100 mg, ~11 µmol, 1.0 eq.) was dissolved in 1 mL argon-purged THF. Triphenylphosphine (29 mg, 0.11 mmol, 10 eq.) was added and the solution was stirred overnight at room temperature under Ar (g) atmosphere. The mixture was diluted with 1 mL THF, followed by addition of 0.4 mL MilliQ water and stirred for 3 hours. The mixture was concentrated under reduced pressure, redissolved in 0.5 mL of CH_2Cl_2, precipitated in cold Et_2O and dried *in vacuo* to obtain NH_2-block copolymer (**10**) as a colorless wax (97 mg, 98%). In the ^{1}H NMR spectrum a singlet was observed at 3.38 ppm, representing the methoxy group, instead of the multiplet that was present in the starting material, representing overlapping signals of the methoxy group and two methylenes of the alkyl group attached to the azide. This indicated full conversion, as the methylene peaks shifted upon reduction of the azide into the amine. Additionally, IR spectroscopy was used to demonstrate the disappearance of the azide signal at 2100 cm^{-1} (Fig. S37).

^{1}H NMR (399 MHz, Chloroform-*d*) δ = 4.39 – 3.98 (m), 3.65 (s), 3.38 (s), 2.44 – 2.26 (m), 2.14 – 1.87 (m), 1.79 – 1.54 (m), 1.48 – 1.32 (m).

Ligand-block copolymer PEG_{22}-*b*-P(CL_{37}-*g*-TMC_{34}-*g*-(TMC-ligand)$_2$) (11)

To a solution of **5** (5.3 mg, 22 µmol, 4 eq.) in 0.4 mL anhydrous CH_2Cl_2/MeCN 1:1 was added a solution of *N*,*N'*-dicyclohexylcarbodiimide (4.6 mg, 22 µmol, 4 eq.) in 0.2 mL anhydrous MeCN. Then, a solution of NH_2-block copolymer (**10**) (50 mg, ~5.6 µmol, 1.0 eq.) and 4-(dimethylamino)pyridine (0.2 mg, 1.6 µmol, 0.3 eq.) in 0.5 mL anhydrous MeCN was added and the mixture was stirred for 2 hours at room temperature under Ar (g) atmosphere. Precipitates were allowed to sediment and the liquid was filtered through a 0.2 µm PTFE syringe filter. The filtrate was concentrated under reduced pressure and the residue was taken up in 5 mL CH_2Cl_2. This was washed once with brine, dried over Na_2SO_4, concentrated under reduced pressure and precipitated in cold Et_2O. It was then dried *in vacuo* to yield ligand-block copolymer (**11**) as a colorless wax (50 mg, 96%). In the ^{1}H NMR spectrum, a shift of the ligand methylene triplets was observed from 2.66 ppm and 2.90 ppm to 2.61 ppm and 2.88 ppm, respectively, indicating conjugation of the ligand to the polymer.

^{1}H NMR (399 MHz, Chloroform-*d*) δ = 7.30 (s), 7.07 – 6.96 (m), 6.80 (d, *J*=8.3), 5.71 (d, *J*=17.8), 5.22 (d, *J*=11.2), 4.49 (hept, *J*=6.0), 4.34 – 3.97 (m), 3.65 (s), 3.38 (s), 2.88 (t, *J*=7.8), 2.61 (t, *J*=7.8), 2.40 – 2.21 (m), 2.13 – 1.85 (m), 1.79 – 1.56 (m), 1.49 – 1.35 (m), 1.34 – 1.29 (m).

HGII-block copolymer PEG_{22}-*b*-P(CL_{37}-*g*-TMC_{34}-*g*-(TMC-HGII)$_2$) (12)

CH_2Cl_2 was purged with Ar (g) for 15 minutes. Ligand-block copolymer (**11**) (50 mg, ~5.4 µmol, 1 eq.) was dissolved 1 mL argon-purged CH_2Cl_2, followed by addition of 2nd generation Grubbs catalyst (11.4 mg, 13.4 µmol, 2.5 eq.) and copper(I) chloride (1.3 mg, 13.4 µmol, 2.5 eq.). The mixture was stirred at 35 °C for 1 hour under Ar (g) atmosphere. It was diluted with 5 mL CH_2Cl_2, washed once with deionised water, dried over Na_2SO_4, concentrated under reduced pressure and precipitated in cold Et_2O to yield HGII-block copolymer (**12**) as a brown wax (43 mg, 88%). On average, 2 catalysts per polymer were attached.

^{1}H NMR (400 MHz, Chloroform-*d*) δ = 16.51 (s), 7.37 – 7.31 (m), 7.17 – 6.99 (m), 6.78 – 6.63 (m), 4.85 (hept, *J*=6.3), 4.44 – 3.99 (m), 3.65 (s), 3.38 (s), 2.91 (t, *J*=7.8), 2.54 (d, *J*=7.8), 2.51 – 2.38 (m), 2.36 – 2.25 (m), 2.10 – 1.93 (m), 1.76 – 1.53 (m), 1.48 – 1.32 (m), 1.31 – 1.23 (m).

Fluorescently labeled block copolymer PEG_{22}-*b*-P(CL_{35}-*g*-TMC_{32})-TMC-Bodipy FL (13) was synthesized according to literature procedure.[5]

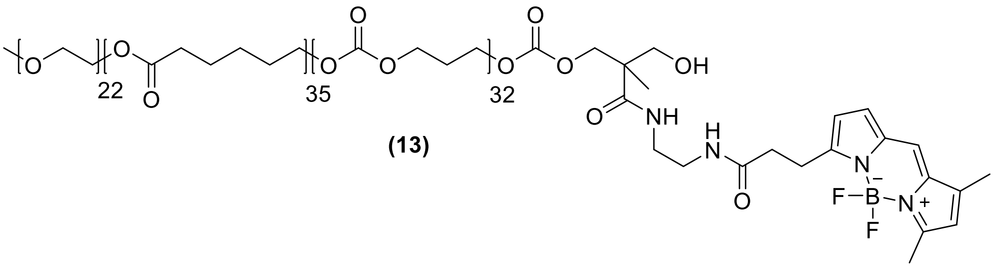

(13)

^{1}H NMR (399 MHz, Chloroform-*d*) δ = 7.09 (s), 6.88 (s), 6.27 (s), 6.12 (s), 4.44 – 3.93 (m), 3.66 (s), 3.39 (s), 3.29 (t, *J*=7.9), 2.77 (t, *J*=7.9), 2.56 (s), 2.38 – 2.23 (m), 2.13 – 1.90 (m), 1.76 – 1.54 (m), 1.50 – 1.31 (m).

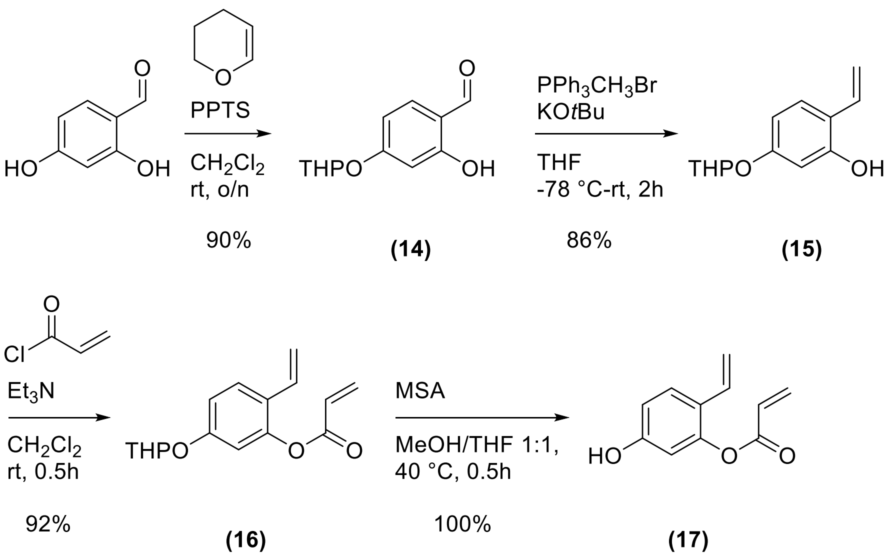

Scheme S5. *Synthesis of ring-closing metathesis substrate (**17**).*

2-Hydroxy-4-[(tetrahydro-2*H*-pyran-2-yl)oxy]benzaldehyde (14) was synthesized according to literature procedure with modifications.[6]

To 250 mL anhydrous CH_2Cl_2, 2,4-dihydroxybenzaldehyde (15.0 g, 109 mmol, 1.0 eq.) was added, followed by pyridinium *p*-toluenesulfonate (2.73 g, 10.9 mmol, 0.1 eq.). Then 3,4-dihydro-2*H*-pyran (11.9 mL, 130 mmol, 1.2 eq.) was added dropwise and the mixture was stirred for 4 hours at room temperature under Ar (g) atmosphere. Additional 3,4-dihydro-2*H*-pyran (5.0 mL, 55 mmol, 0.5 eq.) was added and the reaction mixture was stirred overnight. Then it was washed with saturated $NaHCO_3$ (aq). The aqueous layer was extracted twice with EtOAc. The organic layers were combined, dried over $MgSO_4$ and concentrated under reduced pressure. The resulting colorless oil was purified by flash chromatography (silica gel, heptane/EtOAc gradient 95:5 - 88:12) to give **14** as a white powder (21.7 g, 98 mmol, 90%).

^{1}H NMR (399 MHz, Chloroform-*d*) δ = 11.36 (s, 1H), 9.73 (s, 1H), 7.44 (d, *J*=8.5, 1H), 6.71 – 6.57 (m, 2H), 5.50 (t, *J*=3.2, 1H), 3.91 – 3.77 (m, 1H), 3.69 – 3.56 (m, 1H), 2.10 – 1.50 (m, 6H).
^{13}C NMR (100 MHz, Chloroform-*d*) δ = 194.53, 164.31, 164.13, 135.26, 115.73, 109.38, 103.64, 96.21, 62.17, 29.94, 24.95, 18.42.
GC-EI-MS [M] $^{\bullet+}$ calcd. 222 Da, found 222 Da.

5-((tetrahydro-2H-pyran-2-yl)oxy)-2-vinylphenol (15) was synthesized according to literature procedure with modifications.[7]

To a suspension of methyltriphenylphosphonium bromide (24.1 g, 67.5 mmol, 1.5 eq.) in 90 mL anhydrous THF was added 12% potassium *tert*-butoxide in THF (135 mL, 135 mmol, 3.0 eq.). The mixture was stirred under Ar (g) atmosphere for 1 hour and then cooled to -78 °C. A solution of **14** (10.0 g, 45 mmol, 1.0 eq.) in 110 mL anhydrous THF was added dropwise to the yellow suspension. The

mixture was allowed to warm to room temperature and stirred for another 0.5 hour under Ar (g) atmosphere, after which it was quenched with 730 mL saturated NH_4Cl (aq). The aqueous layer was extracted twice with EtOAc. The organic layers were combined and washed with brine, dried over Na_2SO_4 and concentrated under reduced pressure to give an orange oil. This was purified by flash chromatography (silica gel, heptane/EtOAc gradient 8:2-6:4) and subsequently crystallized from EtOAc/heptane to obtain **15** as a white powder (8.55 g, 38.8 mmol, 86%).

^{1}H NMR (400 MHz, Chloroform-*d*) δ = 7.29 (d, *J*=8.5, 1H), 6.86 (dd, *J*=17.7, 11.2, 1H), 6.61 (dd, *J*=8.5, 2.4, 1H), 6.54 (d, *J*=2.4, 1H), 5.62 (dd, *J*=17.7, 1.4, 1H), 5.39 (t, *J*=3.2, 1H), 5.36 – 5.29 (m, 1H), 5.24 (dd, *J*=11.2, 1.4, 1H), 3.95 – 3.83 (m, 1H), 3.66 – 3.54 (m, 1H), 2.06 – 1.92 (m, 1H), 1.91 – 1.78 (m, 2H), 1.76 – 1.53 (m, 3H).
^{13}C NMR (100 MHz, Chloroform-*d*) δ = 157.62, 153.85, 131.19, 127.98, 118.62, 113.67, 109.36, 103.88, 96.32, 62.01, 30.26, 25.14, 18.59.
GC-EI-MS $[M]^{\bullet+}$ calcd. 220 Da, found 220 Da.

5-((tetrahydro-2H-pyran-2-yl)oxy)-2-vinylphenyl acrylate (16) was synthesized according to literature procedure with modifications.[7]

To a solution of **15** (1.00 g, 4.54 mmol, 1.0 eq.) in anhydrous CH_2Cl_2 was added dropwise triethylamine (1.27 mL, 9.08 mmol, 2.0 eq.) under Ar (g) atmosphere. To this was added dropwise acryloyl chloride (533 µL, 6.81 mmol, 1.5 eq.) and the mixture was stirred for 0.5 hour at room temperature under Ar (g) atmosphere. Then it was concentrated under reduced pressure and purified by flash chromatography (silica gel, heptane/EtOAc 8:1) to give **16** as a white solid (1.14 g, 4.16 mmol, 92%), which was found stable when stored at -30 °C.

^{1}H NMR (399 MHz, Chloroform-*d*) δ = 7.49 (d, *J*=8.7, 1H), 6.94 (dd, *J*=8.7, 2.5, 1H), 6.81 (d, *J*=2.5, 1H), 6.73 – 6.55 (m, 2H), 6.34 (dd, *J*=17.3, 10.4, 1H), 6.04 (dd, *J*=10.4, 1.2, 1H), 5.63 (dd, *J*=17.5, 1.2, 1H), 5.42 (t, *J*=3.2, 1H), 5.20 (dd, *J*=11.1, 1.2, 1H), 3.97 – 3.79 (m, 1H), 3.70 – 3.50 (m, 1H), 2.07 – 1.91 (m, 1H), 1.91 – 1.78 (m, 2H), 1.77 – 1.51 (m, 3H).
^{13}C NMR (100 MHz, Chloroform-*d*) δ = 164.20, 157.43, 148.47, 132.81, 129.91, 127.66, 126.96, 123.62, 114.68, 114.30, 110.46, 96.42, 61.88, 30.20, 25.15, 18.52.
MALDI-TOF MS $[M+Na]^+$ calcd. 297.11 Da, found 297.10 Da.

5-hydroxy-2-vinylphenyl acrylate (17)

To a solution of **16** (0.10 g, 0.36 mmol, 1.0 eq.) in 6.0 mL MeOH/THF 1:1 was added methanesulfonic acid (3.1 uL, 0.047 mmol, 0.13 eq.) and the mixture was stirred at 40 °C for 30 minutes. Then it was concentrated under reduced pressure to approximately 5 mL. It was not fully concentrated, as the product is not stable in its neat form due to polymerization. The solution was diluted with 25 mL EtOAc and concentrated to approximately 5 mL again. This was repeated once to remove most of the THF and MeOH. It was then washed three times with brine and dried over Na_2SO_4. Hereafter, 700 µL of deuterated dimethylsulfoxide (DMSO-d_6) was added and the mixture was concentrated under reduced pressure to remove all solvents except for DMSO-d_6 to obtain a 0.58 M transparent stock solution of **17** which was stored at -30 °C (it is stable at room temperature for at least 3 days). The concentration of the stock solution was determined using quantitative ^{1}H NMR using 1,3,5-trimethoxybenzene as internal standard and an increased relaxation delay of 30 seconds.

^{1}H NMR (400 MHz, DMSO-d_6) δ = 9.94 (s, 1H), 7.51 (d, *J*=8.5, 1H), 6.72 (dd, *J*=8.5, 2.5, 1H), 6.63 – 6.48 (m, 3H), 6.42 (dd, *J*=17.3, 10.2, 1H), 6.16 (dd, *J*=10.2, 1.2, 1H), 5.64 (dd, *J*=17.7, 1.2, 1H), 5.15 (dd, *J*=11.1, 1.2, 1H).
^{13}C NMR (100 MHz, DMSO-d_6) δ = 164.36, 158.52, 148.82, 134.25, 130.10, 127.87, 127.55, 120.97, 114.29, 113.87, 109.81.
GC-EI MS $[M]^{\bullet+}$ calcd. 190 Da, found 190 Da.

Umbelliferone (18)

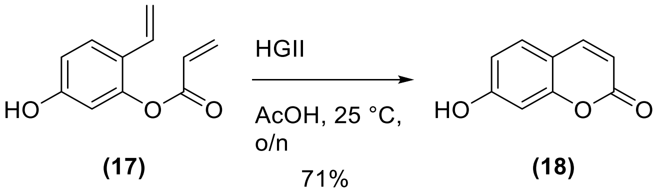

Scheme S6. *Conversion of fluorogenic substrate **17** into umbelliferone (**18**).*

To 30 µL of 540 mM substrate (**17**) in DMSO (16 µmol, 1.0 eq.) was added 0.5 mL of glacial acetic acid, followed by 80 µL of 10 mM HGII in CH_2Cl_2 (0.8 µmol, 0.05 eq.). The solution was stirred at 25 °C overnight. Then 5 mL of heptane was added and the mixture was concentrated under reduced pressure. This was repeated once. Afterwards, the residue was diluted with DMSO-d_6 for ^{1}H NMR analysis of the crude mixture. A conversion of 71% to **18** was determined by integration of the free doublet at 7.93 ppm.

^{1}H NMR (400 MHz, DMSO-d_6) δ = 7.93 (d, *J*=9.5, 1H), 7.53 (d, *J*=8.5, 1H), 6.79 (dd, *J*=8.5, 2.3, 1H), 6.72 (d, *J*=2.2, 1H), 6.20 (d, *J*=9.5, 1H).

***N*,*N*-Diallyl-*p*-toluenesulfonamide (19)** was synthesized according to literature procedure.[8]

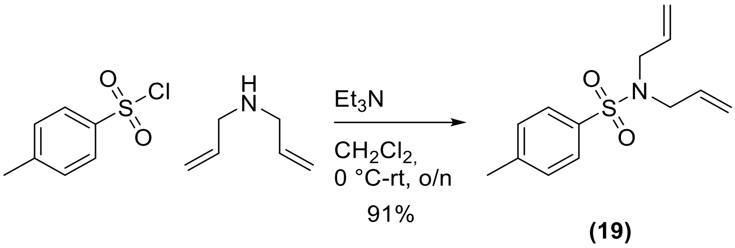

Scheme S7. *Synthesis of ring closing metathesis model substrate (**19**).*

A solution of diallylamine (1.85 mL, 15.0 mmol, 1.2 eq.) in 13 mL of anhydrous CH_2Cl_2 was stirred at 0 °C. Triethylamine (2.09 g, 15 mmol, 1.2 eq.) was added dropwise, followed by dropwise addition of a solution of *p*-toluenesulfonyl chloride (2.38 g, 12.5 mmol, 1.0 eq.) in 12 mL of anhydrous CH_2Cl_2. The solution was stirred overnight while slowly warming to room temperature. Then it was washed once with 1M $KHSO_4$ (aq), once with saturated $NaHCO_3$ (aq) and once with brine, dried over $MgSO_4$ and concentrated under reduced pressure. The resulting yellow oil was purified by flash chromatography (silica gel, heptane/EtOAc 3:1) to obtain **19** as a transparent oil (2.86 g, 11.4 mmol, 91%).

^{1}H NMR (399 MHz, Chloroform-*d*) δ = 7.78 – 7.62 (m, 2H), 7.30 (d, *J*=8.1, 2H), 5.72 – 5.47 (m, 2H), 5.26 – 5.02 (m, 4H), 3.80 (dt, *J*=6.1, 1.3, 4H), 2.43 (s, 3H).
^{13}C NMR (100 MHz, Chloroform-*d*) δ = 143.22, 137.44, 132.67, 129.68, 127.18, 118.96, 49.33, 21.52.
MALDI-TOF MS $[M+Na]^+$ calcd. 274.09 Da, found 274.09 Da.

1-tosyl-2,5-dihydro-1*H*-pyrrole (20)

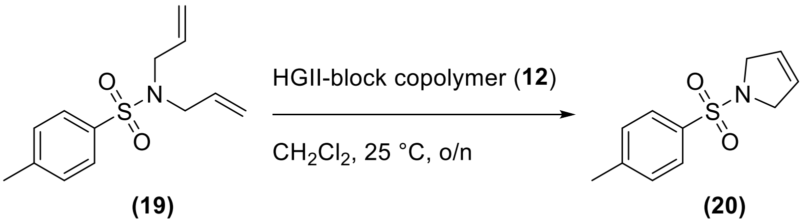

Scheme S8. *Conversion of model substrate **19** as catalyzed by HGII-conjugated copolymer (**12**).*

To a solution of HGII-block copolymer (**12**) (1.2 mg, ~0.12 µmol, 0.01 eq.) in 1.0 mL CH_2Cl_2 was added *N*-tosyldiallylamine (**19**) (3.0 mg, 12 µmol, 1.0 eq.). The solution was stirred overnight at 25 °C under Ar (g) atmosphere. TLC (silica gel, heptane/EtOAc 8:2) showed that full conversion was reached. The

mixture was then concentrated under reduced pressure to a small volume and precipitated in 1 mL cold Et_2O. The precipitate was separated from the liquid and the latter was concentrated under reduced pressure. The resulting residue was analyzed by ^1H NMR spectroscopy to observe full conversion to **20** as no starting material was present anymore.

^1H NMR (400 MHz, Chloroform-*d*) δ = 7.72 (d, *J*=8.1, 2H), 7.32 (d, *J*=8.1, 2H), 5.65 (s, 2H), 4.12 (s, 4H), 2.43 (s, 3H).

Fluorescence calibration curve of umbelliferone

A concentration range of umbelliferone (99% purity, Sigma-Aldrich) in PBS was prepared: 0 µM, 0.20 µM, 0.40 µM, 1.0 µM, 2.0 µM, 4.0 µM, 8.0 µM, 14.0 µM. To 50 µL of each solution was added 5 µL DMSO, 20 µL PBS and 25 µL HGII-loaded polymersomes (10 mg mL^{-1} in PBS). Fluorescence of the samples was measured using a microplate reader ($\lambda_{Ex}/\lambda_{Em}$ = 322 nm/440 nm).

Determination of ruthenium and copper content in polymersome samples by ICP-MS

Two batches of covalent and non-covalent loaded HGII-polymersomes in PBS were prepared via direct hydration (10 mg mL^{-1}). Of each type, one was filtered using a 0.45 µm PVDF syringe filter and the other was not. To 20 µL of sample (triplo) was added 160 µL of 65% HNO_3 (aq). Then, the samples were heated at 80 °C for 1 hour after which they were diluted to 10 mL with MilliQ water. Samples were analyzed for ruthenium content by ICP-MS analysis (theoretical Ru content: 80 ppb).

	Non-filtered	*Filtered over 0.45 µm PVDF*
Covalent	84.68 ± 4.95 ppb	56.68 ± 3.56 ppb
Non-covalent	71.98 ± 1.78 ppb	52.28 ± 0.95 ppb

Table S1. *Ruthenium content of nanoreactors determined by ICP-MS analysis before and after filtration over a 0.45 µm PVDF membrane.*

Copper content of filtered covalent-loaded HGII-polymersomes samples was 6.00 ± 0.35 ppb, indicating that most of the CuCl used in the last step of the polymer preparation was removed during the work-up.

Calculation of expected amount of ruthenium

A typical batch of 2 mg polymersomes contains 0.4 mg of HGII-conjugated copolymer (20 wt%). The HGII-copolymer has an average molecular weight of 10.3 kDa and contains on average 2 catalysts per polymer. A batch of 2 mg polymersomes therefore contains 80 nmol of catalyst (=40 nmol mg^{-1}). The ICP-MS samples prepared with 20 µL of a 10 mg mL^{-1} polymersome dispersion contain a theoretical 8 nmol of ruthenium. These samples were diluted to 10 mL, corresponding to an expected final ruthenium content of 80 ppb.

3. Supplementary figures and tables

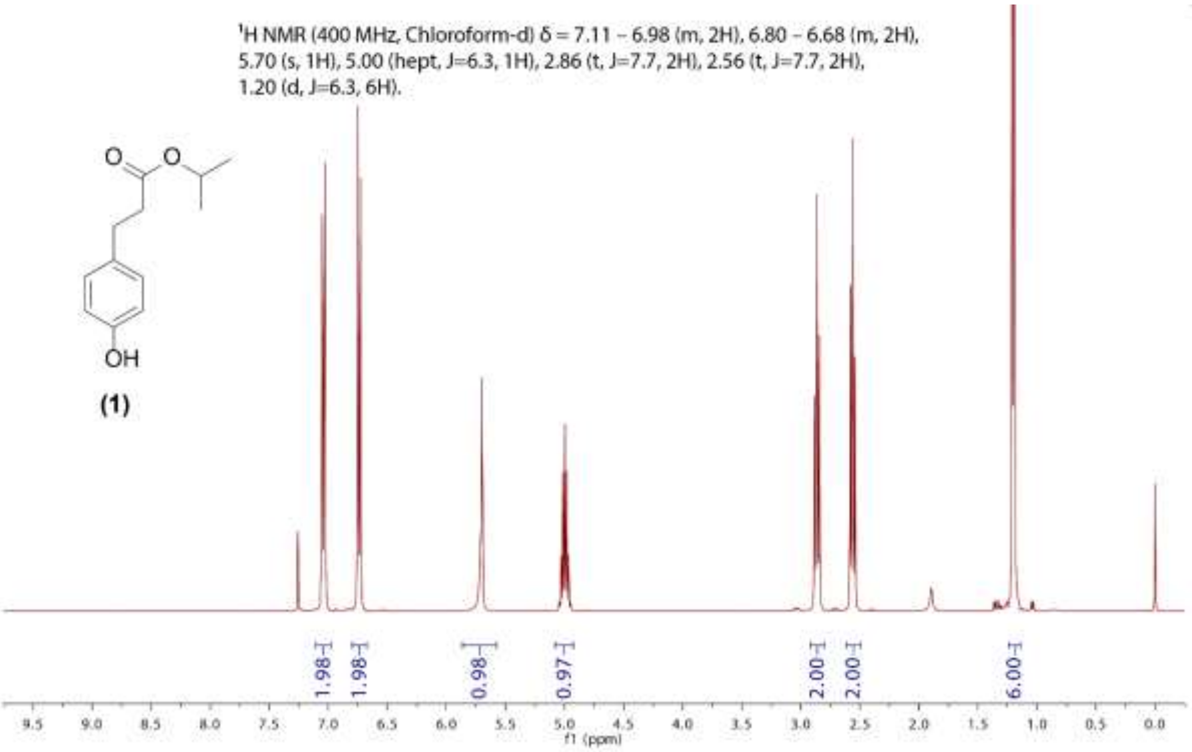

Figure S1. *^{1}H NMR spectrum of* ***1****.*

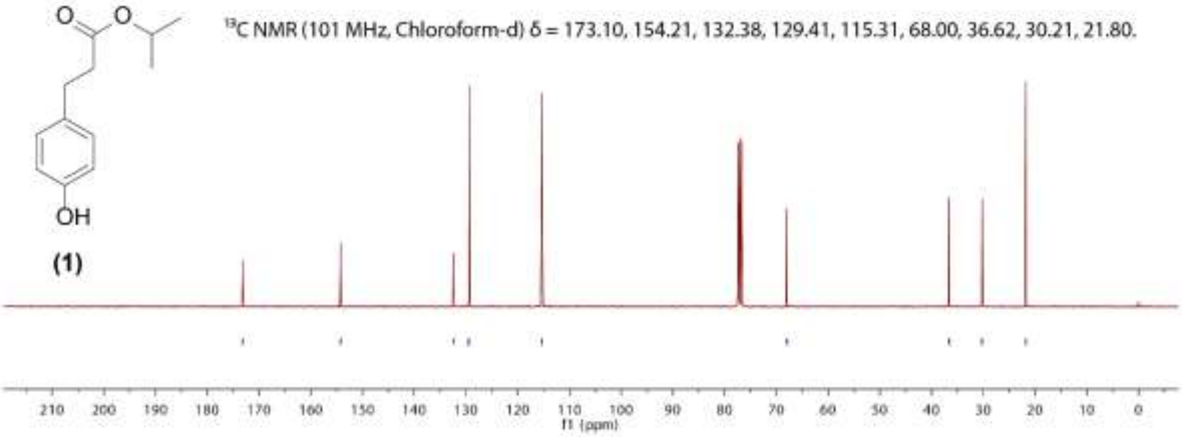

Figure S2. *^{13}C NMR spectrum of* ***1****.*

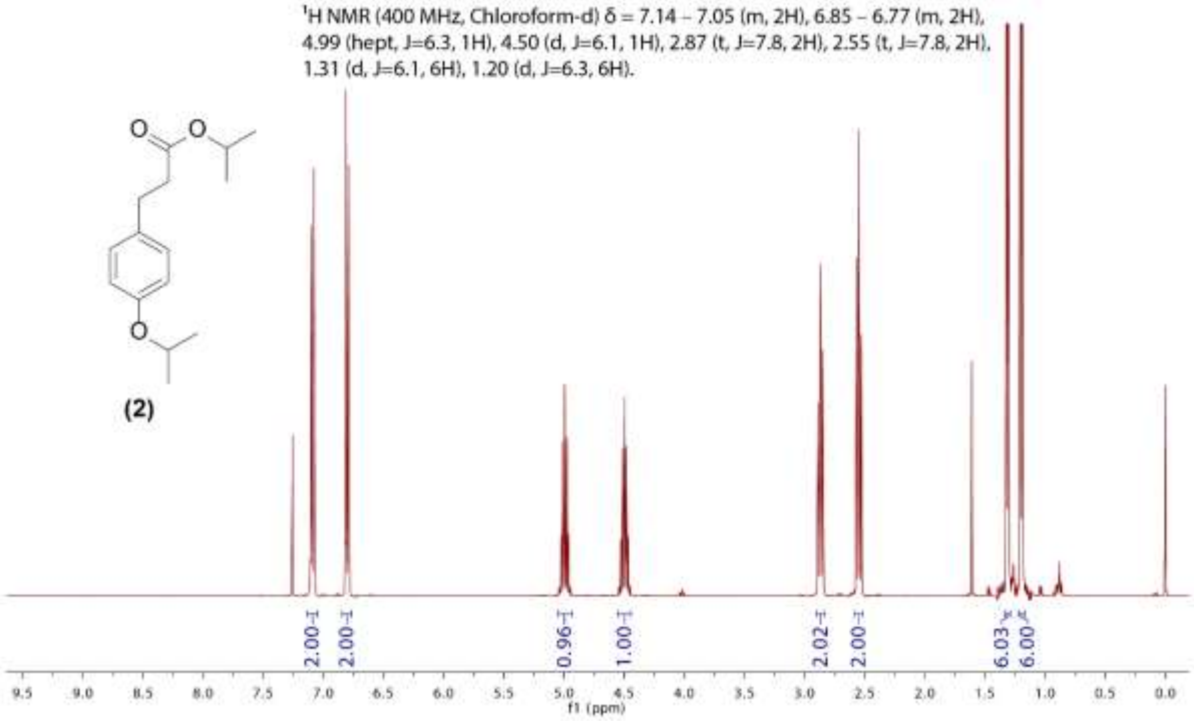

Figure S3. *^{1}H NMR spectrum of* ***2****.*

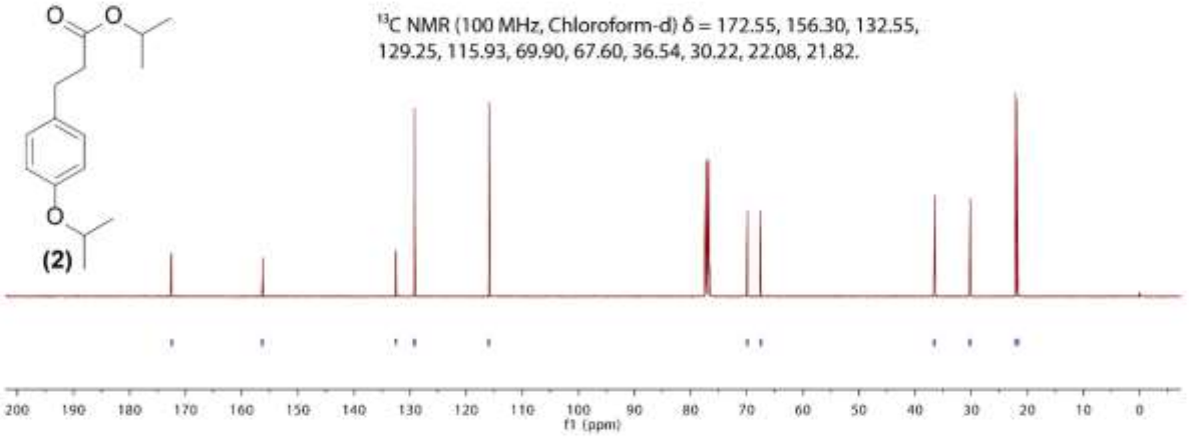

Figure S4. *^{13}C NMR spectrum of* ***2****.*

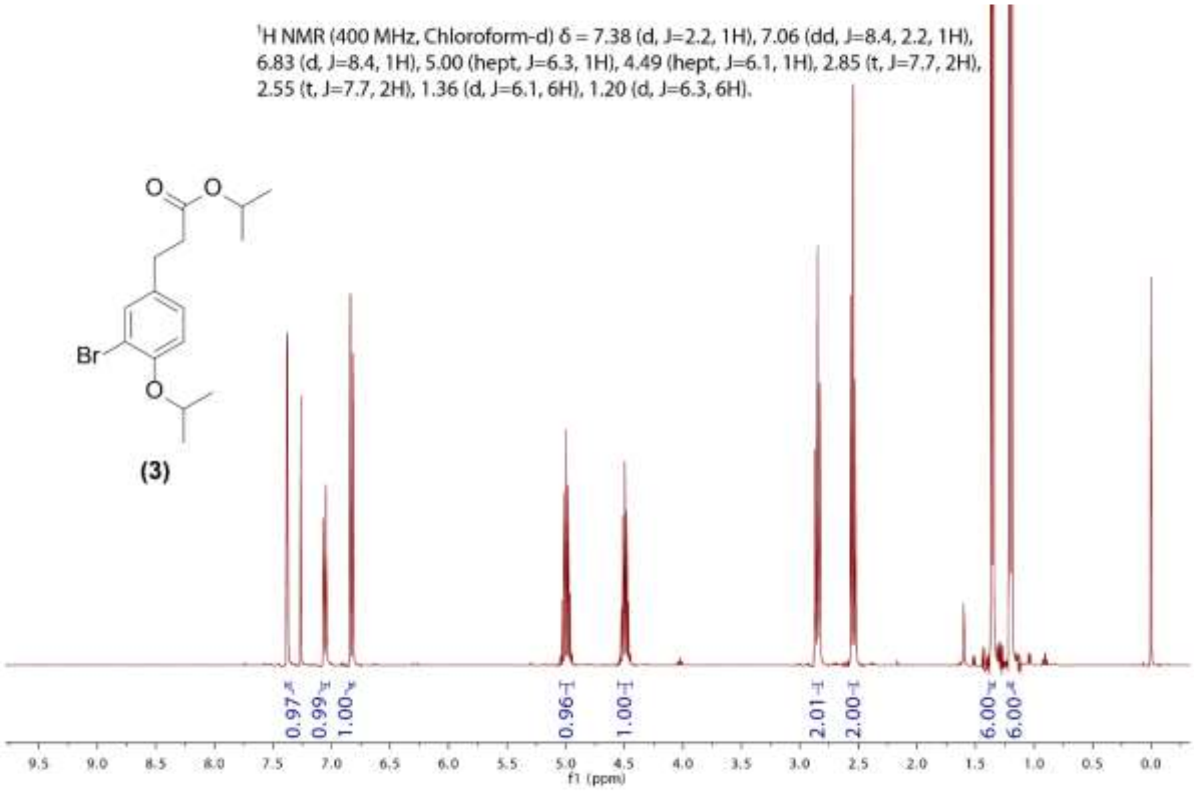

Figure S5. *^{1}H NMR spectrum of **3**.*

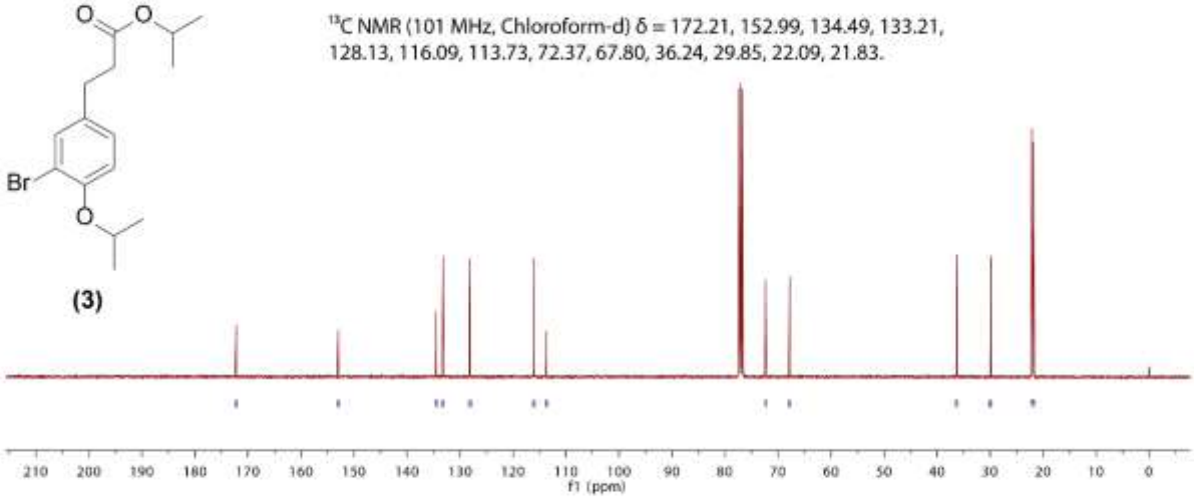

Figure S6. *^{13}C NMR spectrum of **3**.*

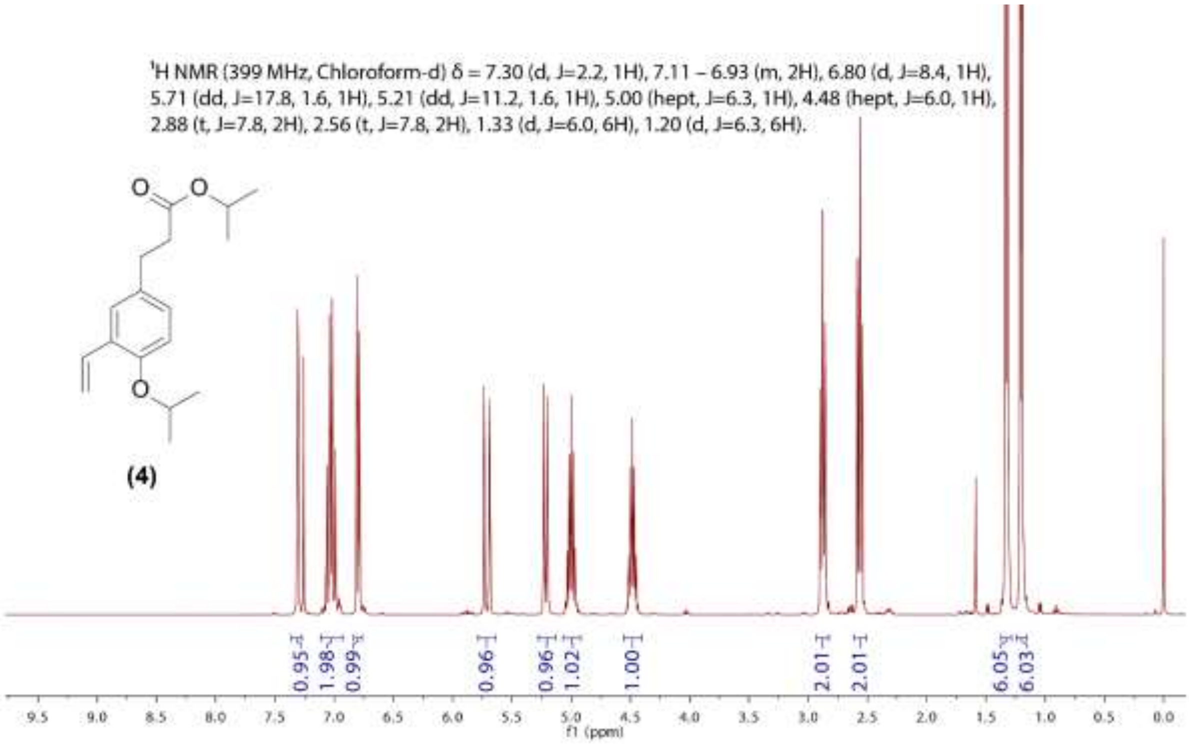

Figure S7. *¹H NMR spectrum of **4**.*

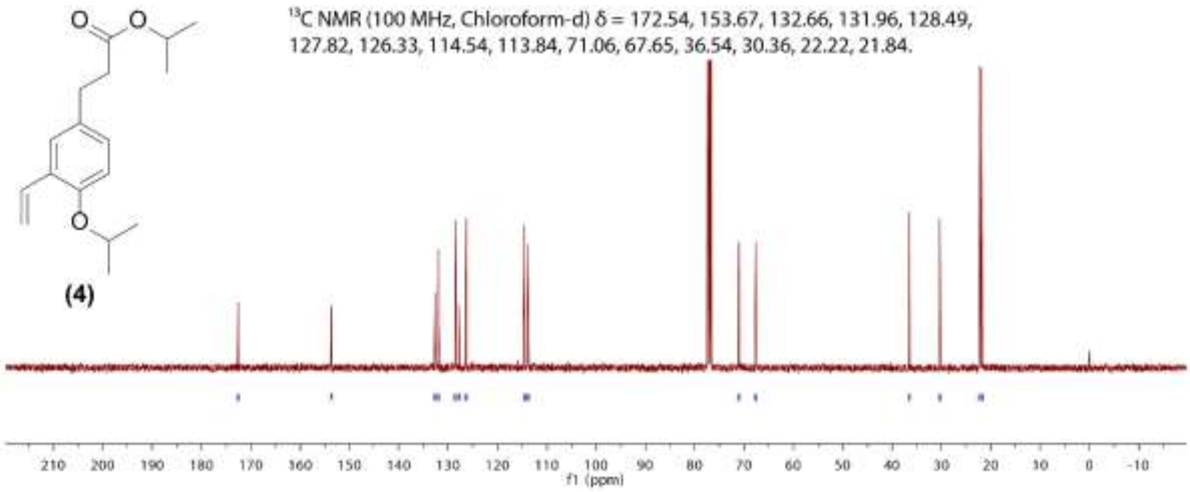

Figure S8. *¹³C NMR spectrum of **4**.*

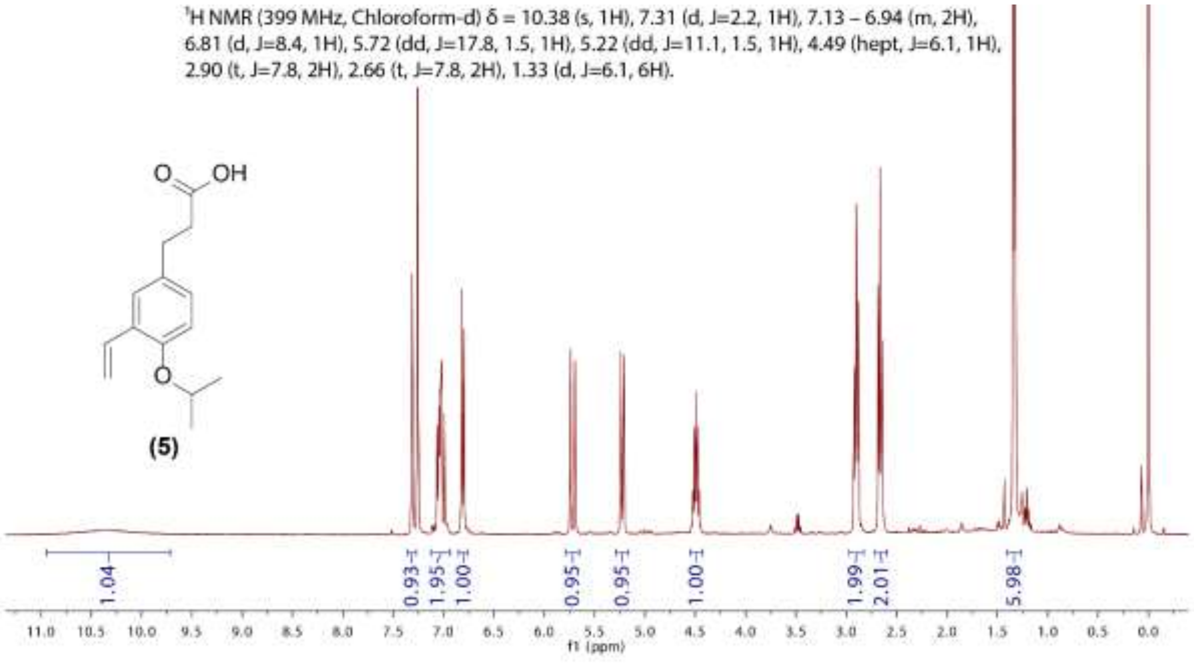

Figure S9. *^{1}H NMR spectrum of **5**.*

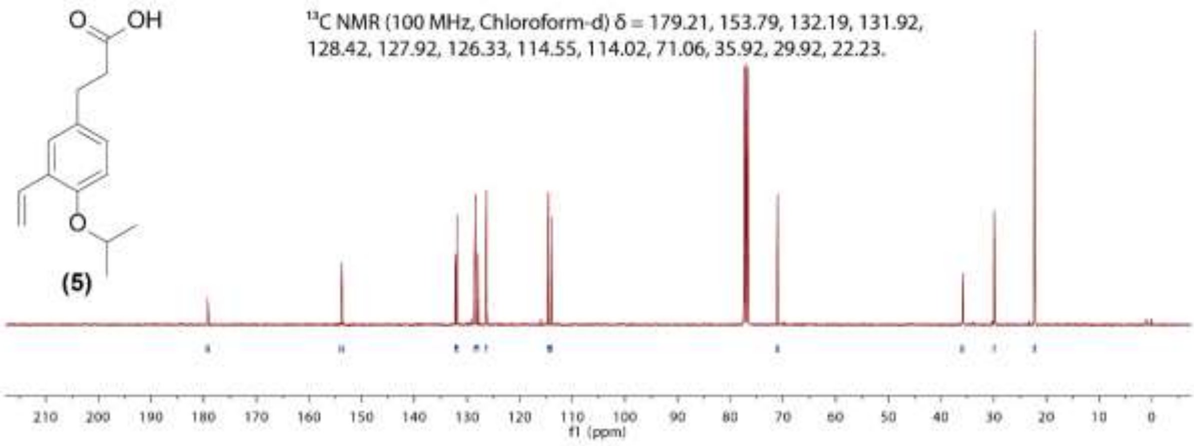

Figure S10. *^{13}C NMR spectrum of **5**.*

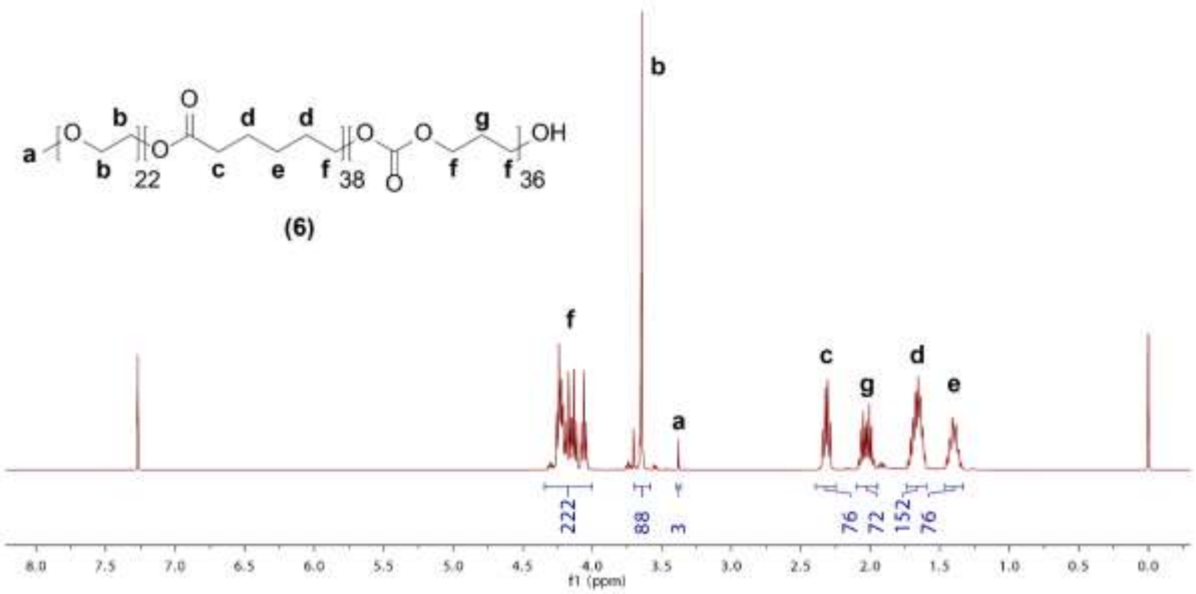

Figure S11. ^{1}*H NMR spectrum of PEG$_{22}$-b-P(CL$_{38}$-g-TMC$_{36}$)* (***6***).

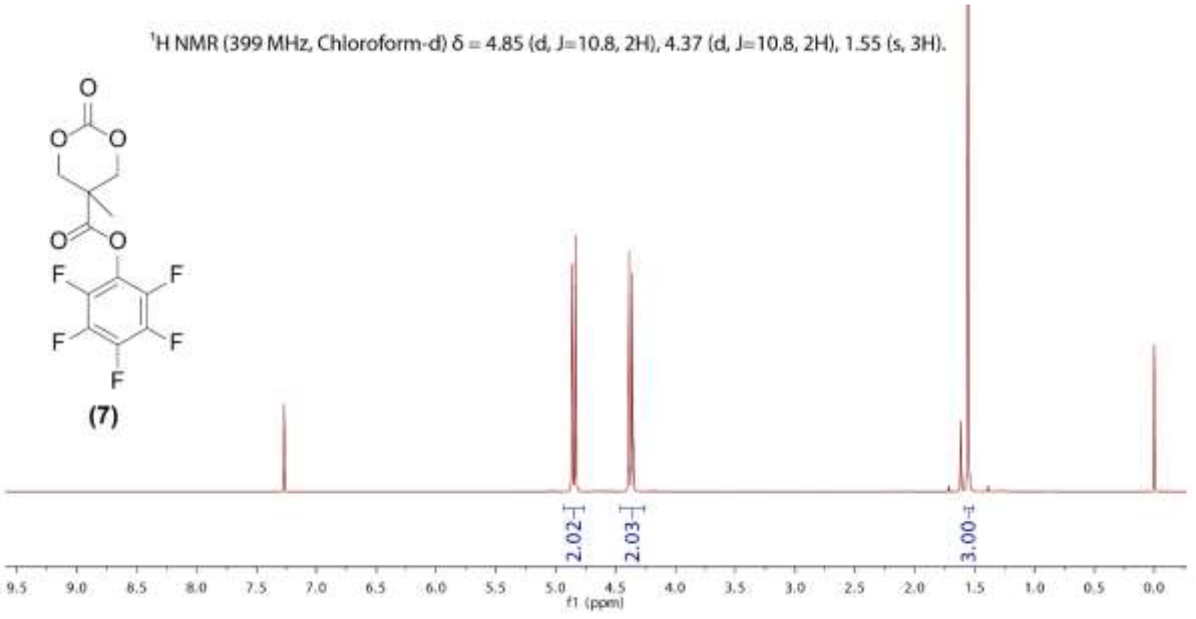

Figure S12. ^{1}*H NMR spectrum of* ***7****.*

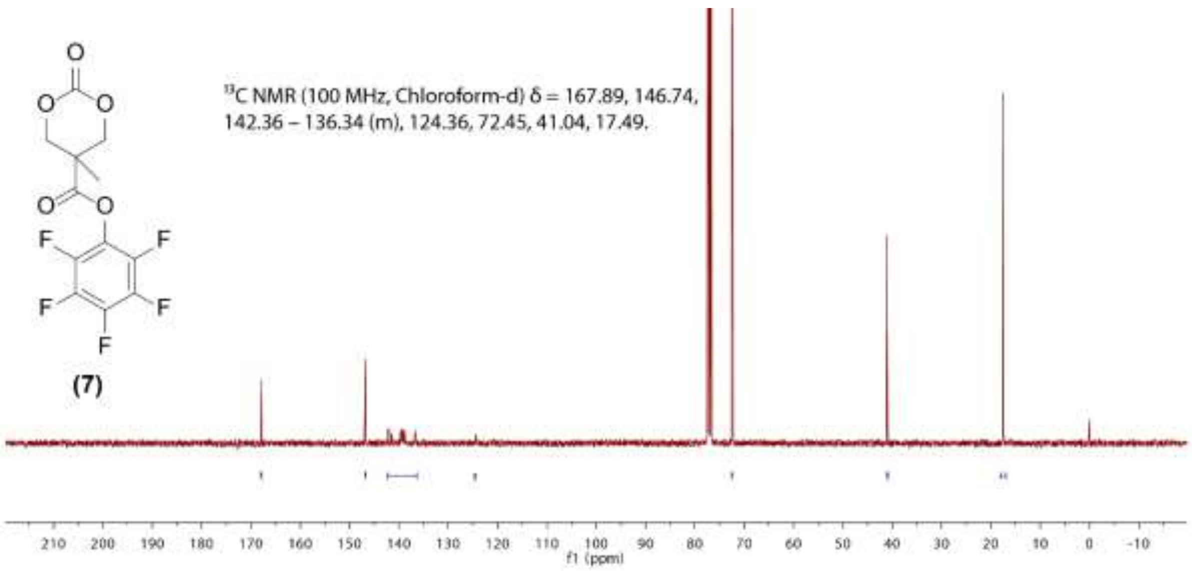

Figure S13. *^{13}C NMR spectrum of **7**.*

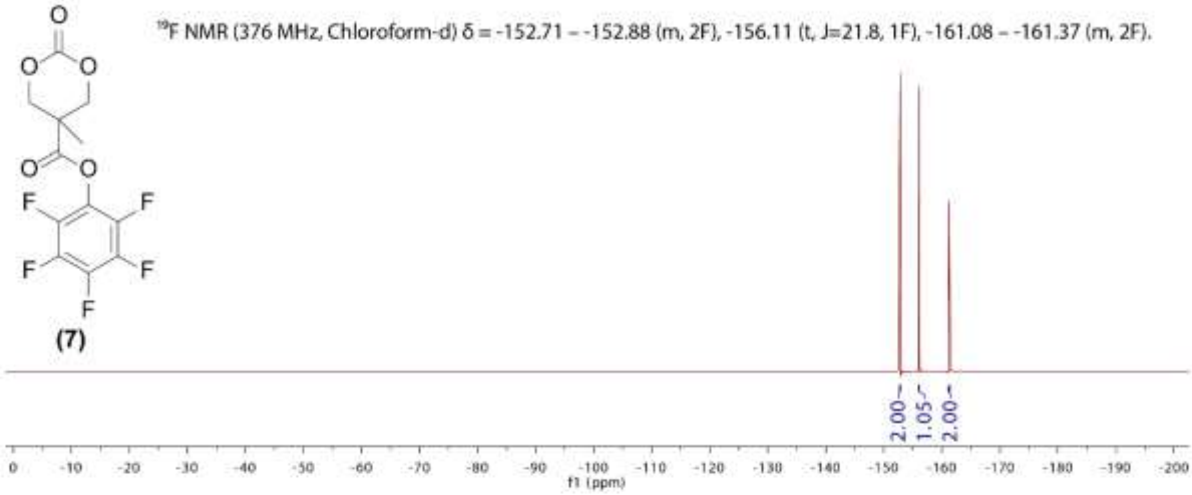

Figure S14. *^{19}F NMR spectrum of **7**.*

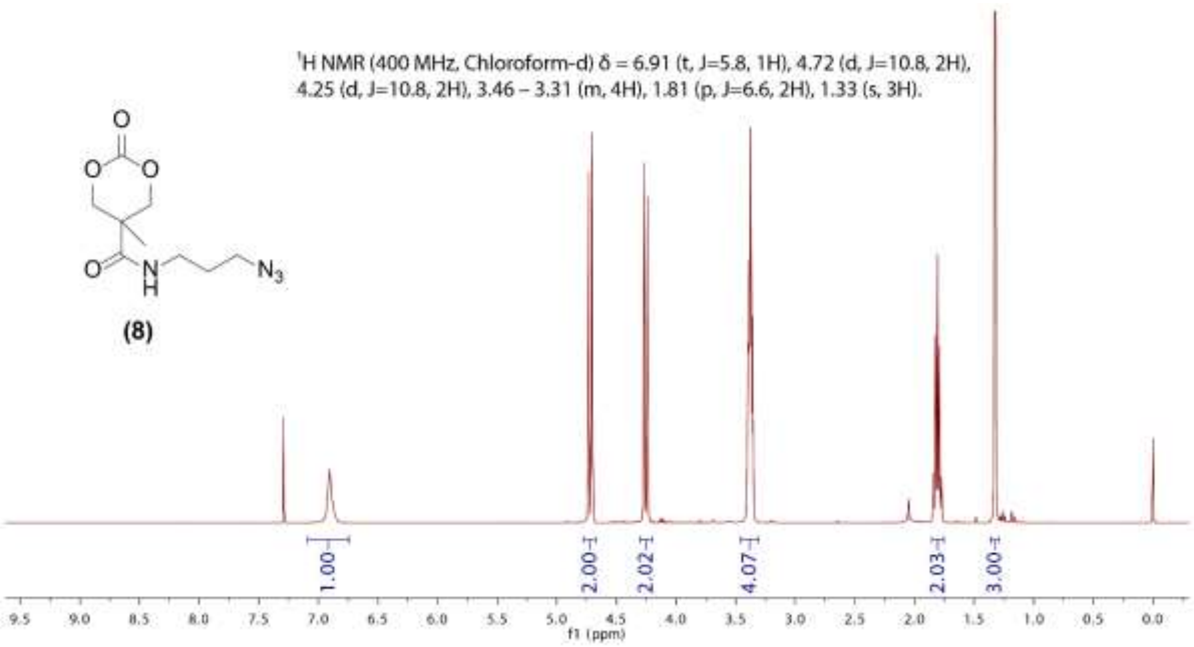

Figure S15. *^{1}H NMR spectrum of **8**.*

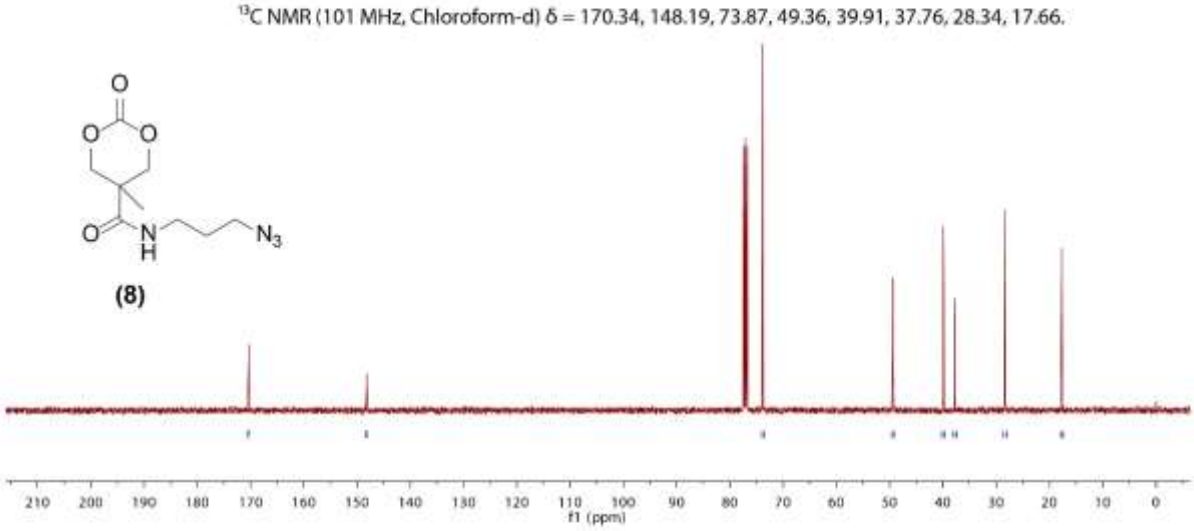

Figure S16. *^{13}C NMR spectrum of **8**.*

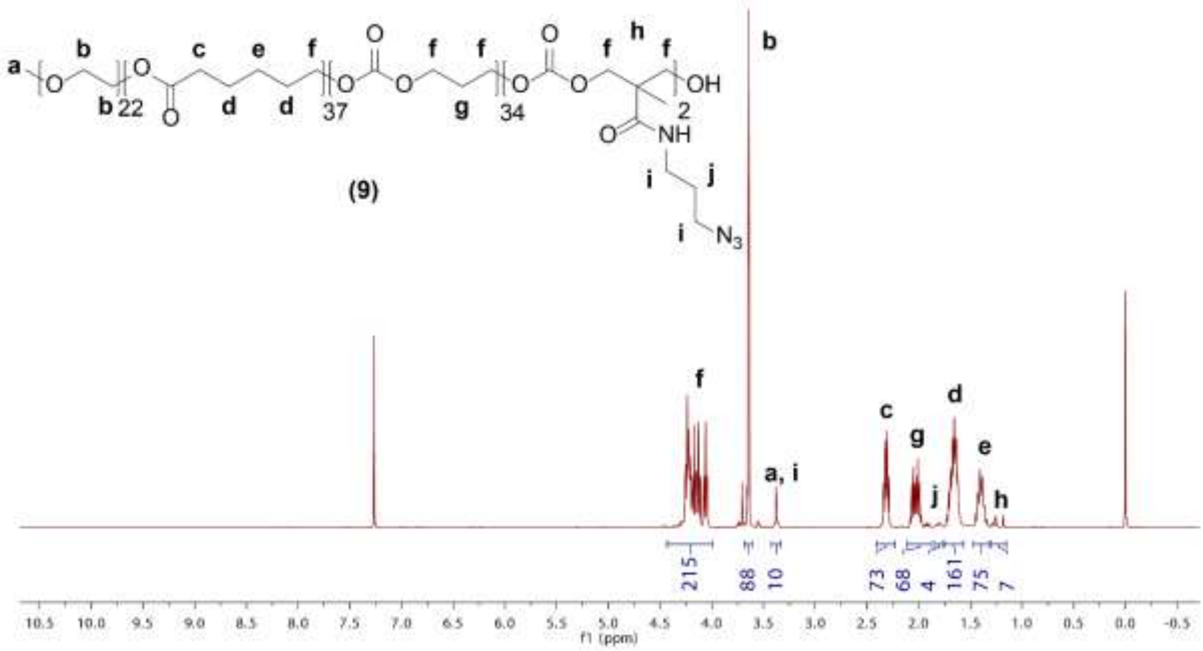

Figure S17. *^{1}H NMR spectrum of N_3-block copolymer (**9**).*

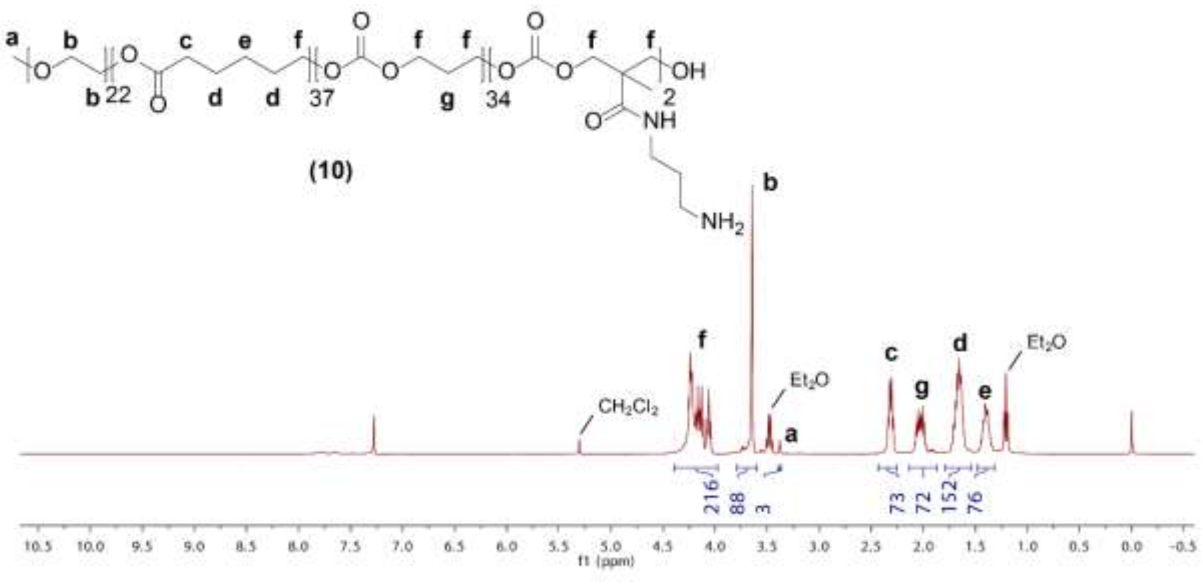

Figure S18. *^{1}H NMR spectrum of NH_2-block copolymer (**10**).*

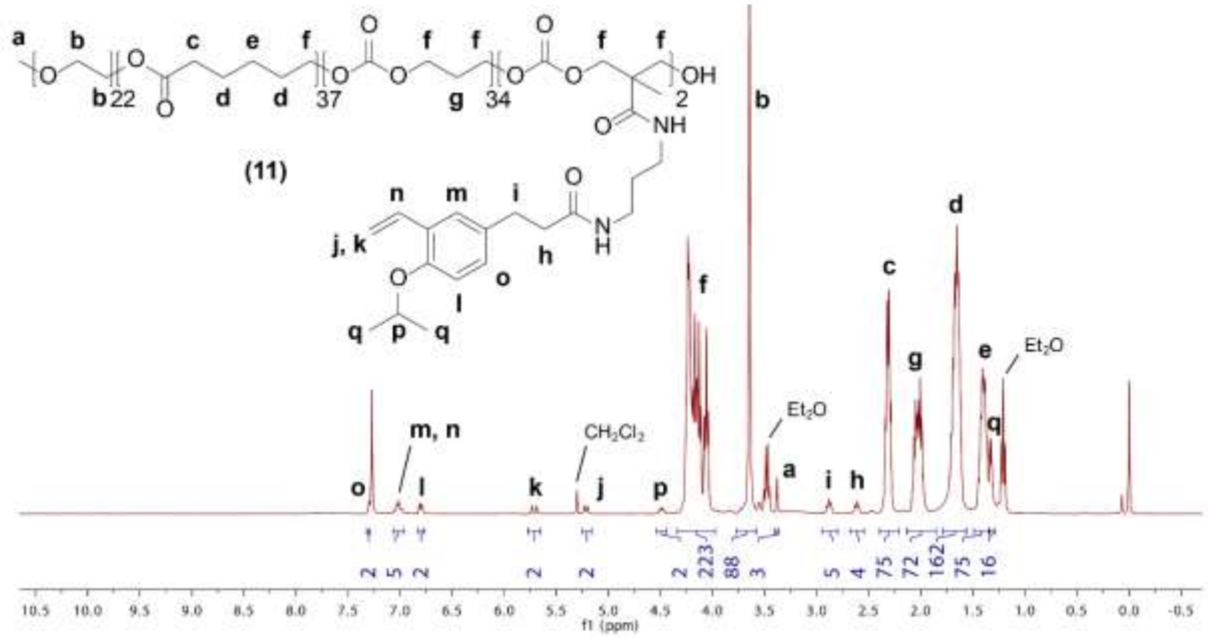

Figure S19. ^{1}*H NMR spectrum of ligand-block copolymer (**11**).*

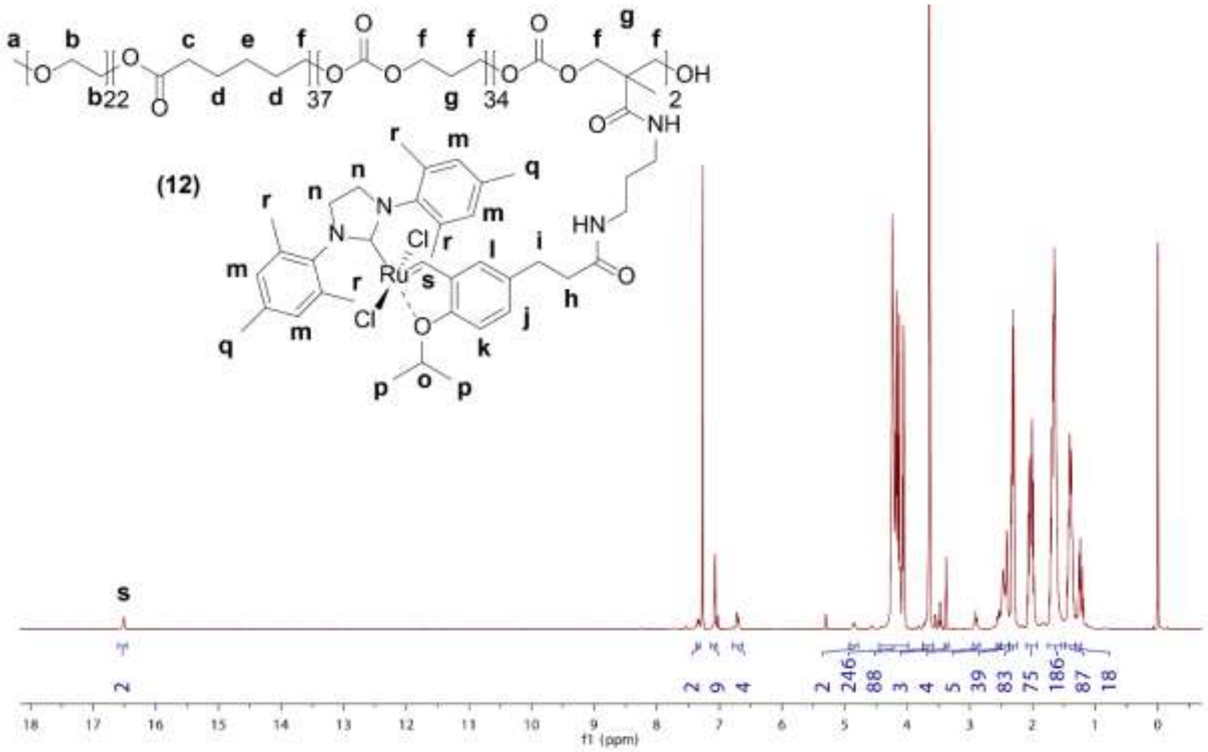

Figure S20. ^{1}*H NMR spectrum of HGII-block copolymer (**12**). See figure S21 for the allocation of the other signals.*

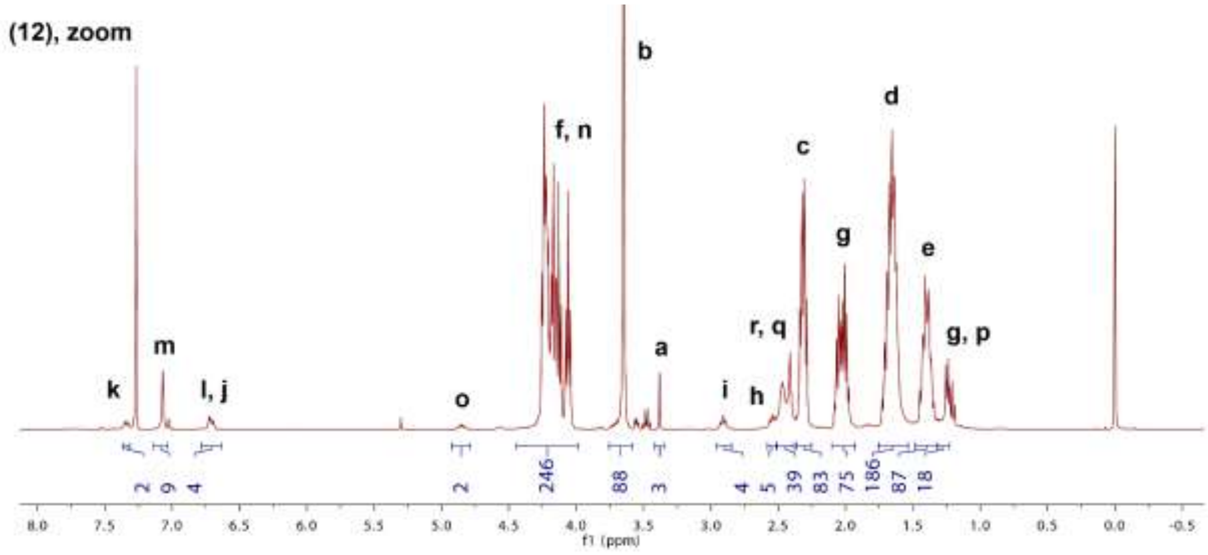

Figure S21. *Zoom of ^{1}H NMR spectrum of HGII-block copolymer (**12**).*

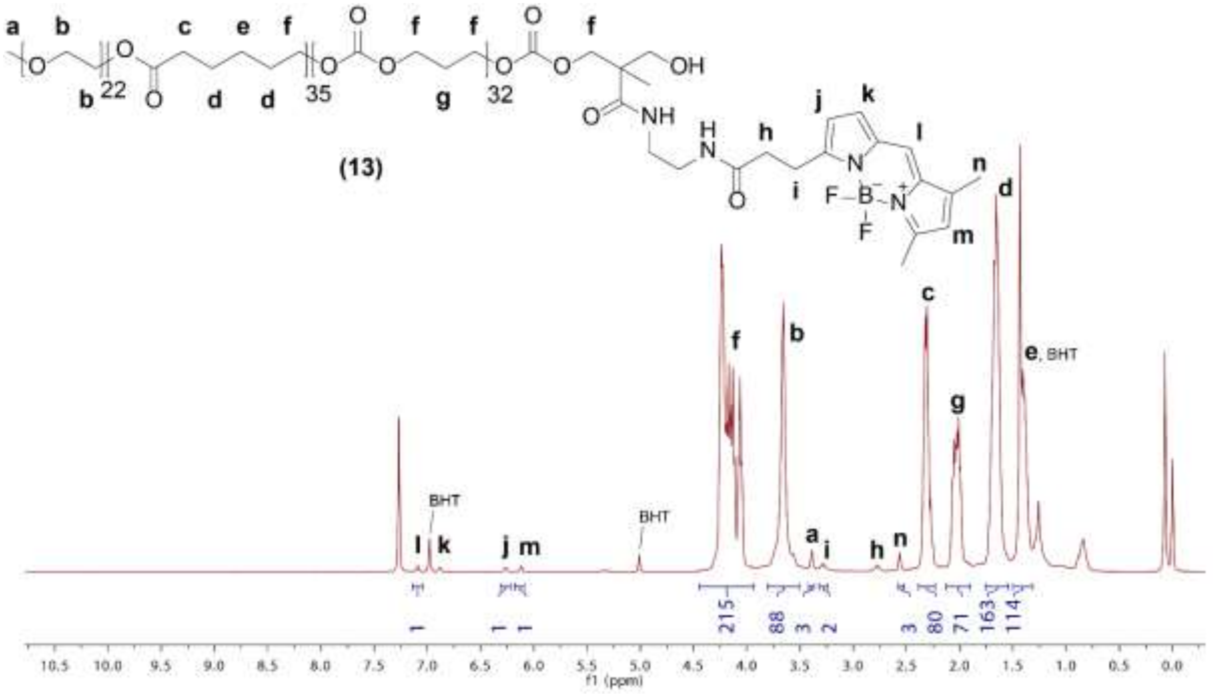

Figure S22. *^{1}H NMR spectrum of Bodipy FL-block copolymer (**13**).*[5]

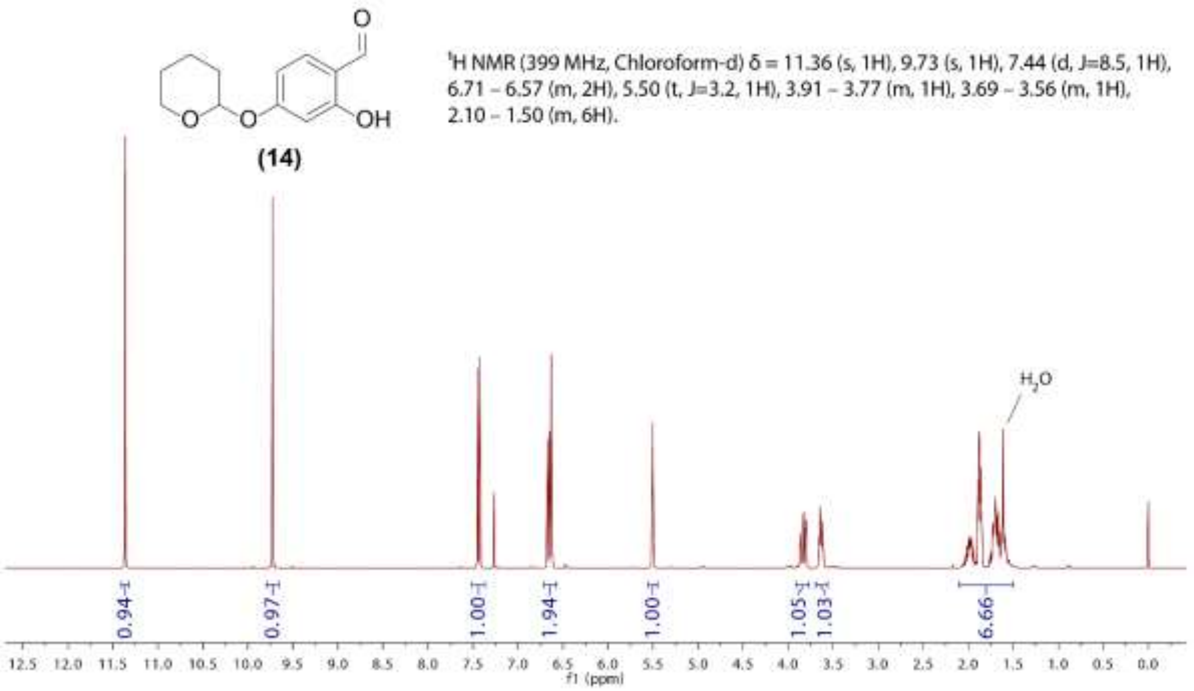

Figure S23. *^{1}H NMR spectrum of* ***14****.*

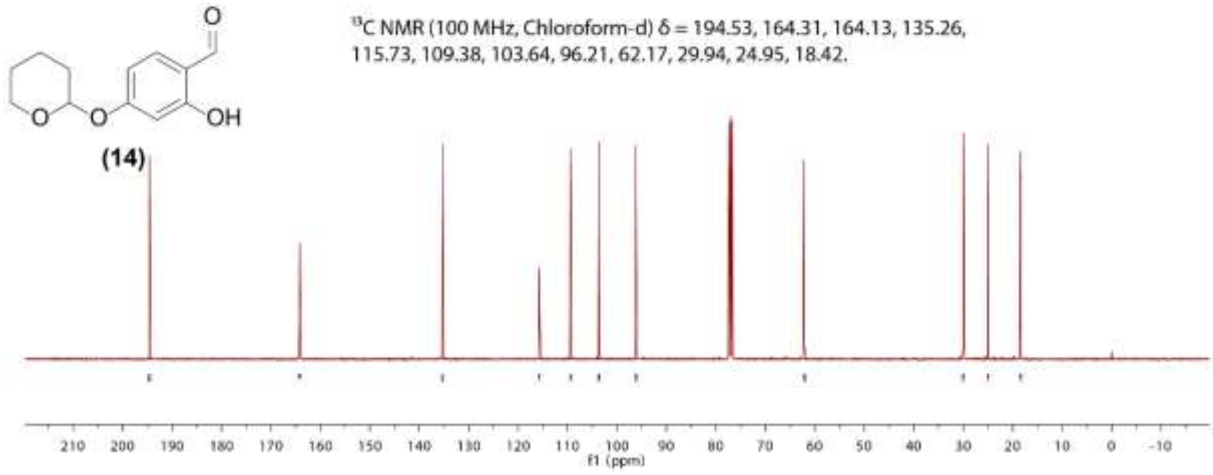

Figure S24. *^{13}C NMR spectrum of* ***14****.*

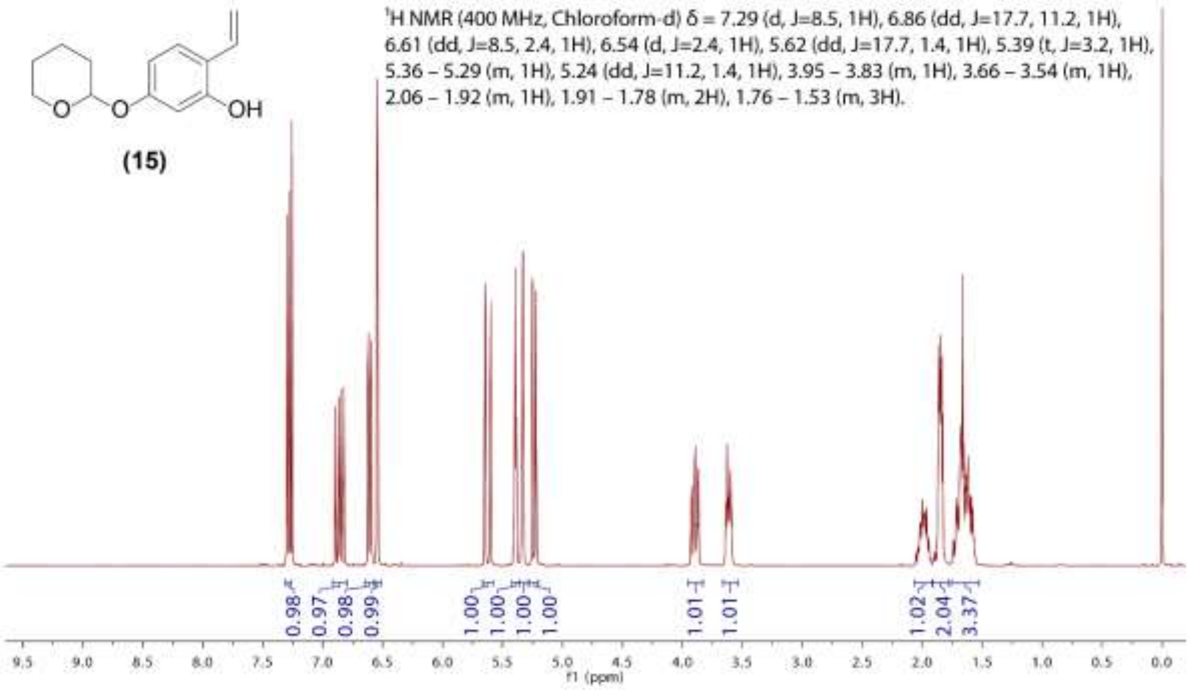

Figure S25. *^{1}H NMR spectrum of* ***15****.*

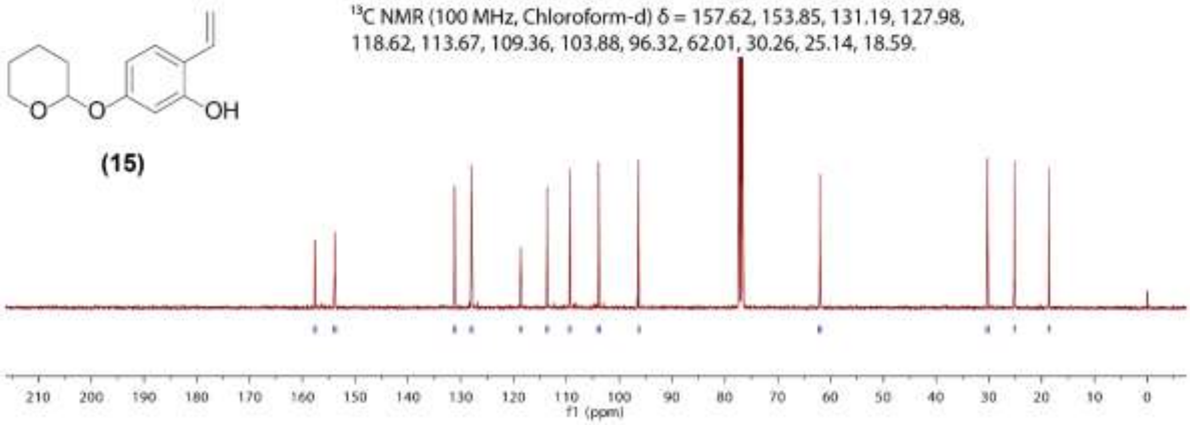

Figure S26. *^{13}C NMR spectrum of* ***15****.*

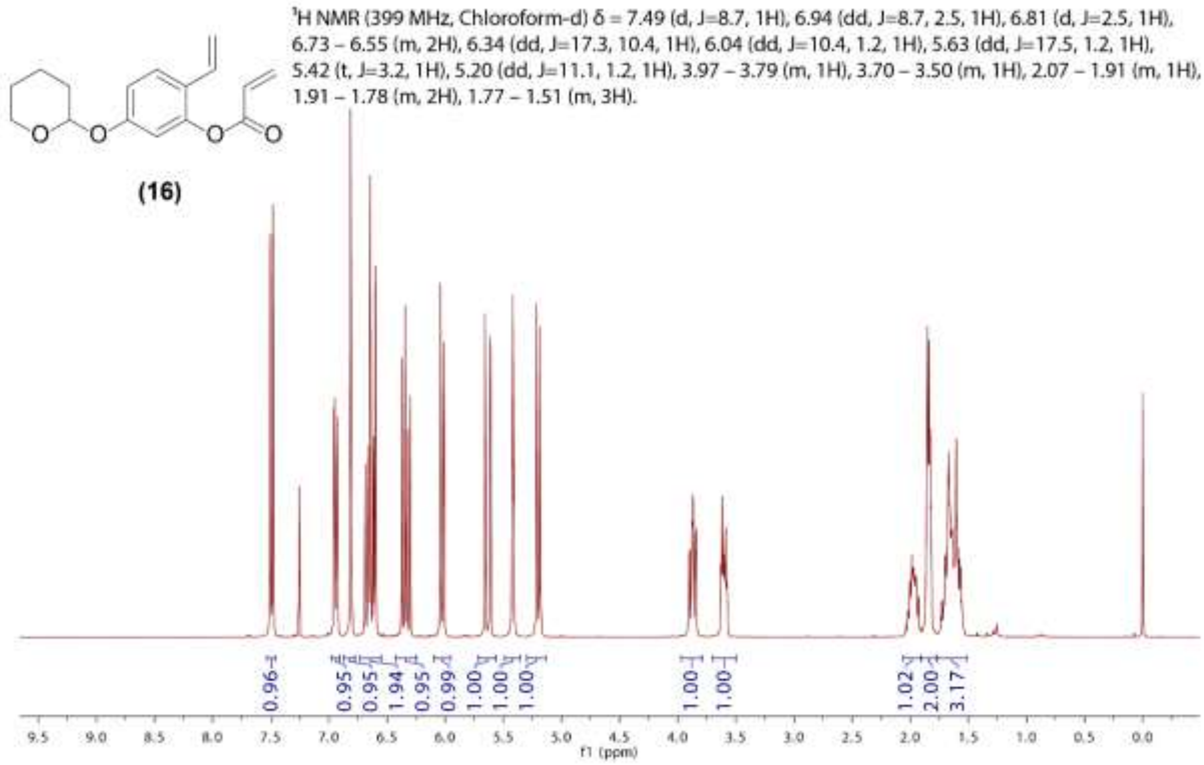

Figure S27. *^{1}H NMR spectrum of **16**.*

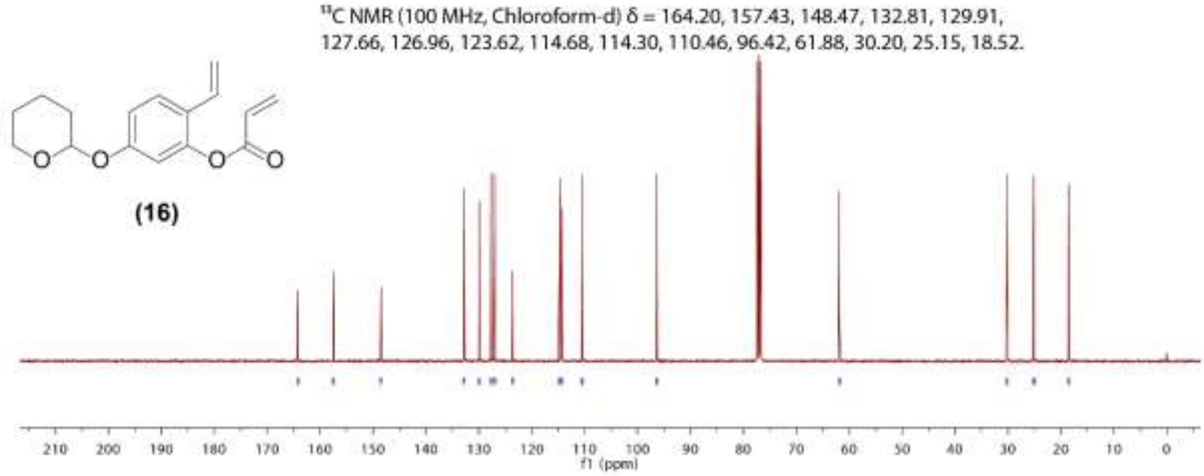

Figure S28. *^{13}C NMR spectrum of **16**.*

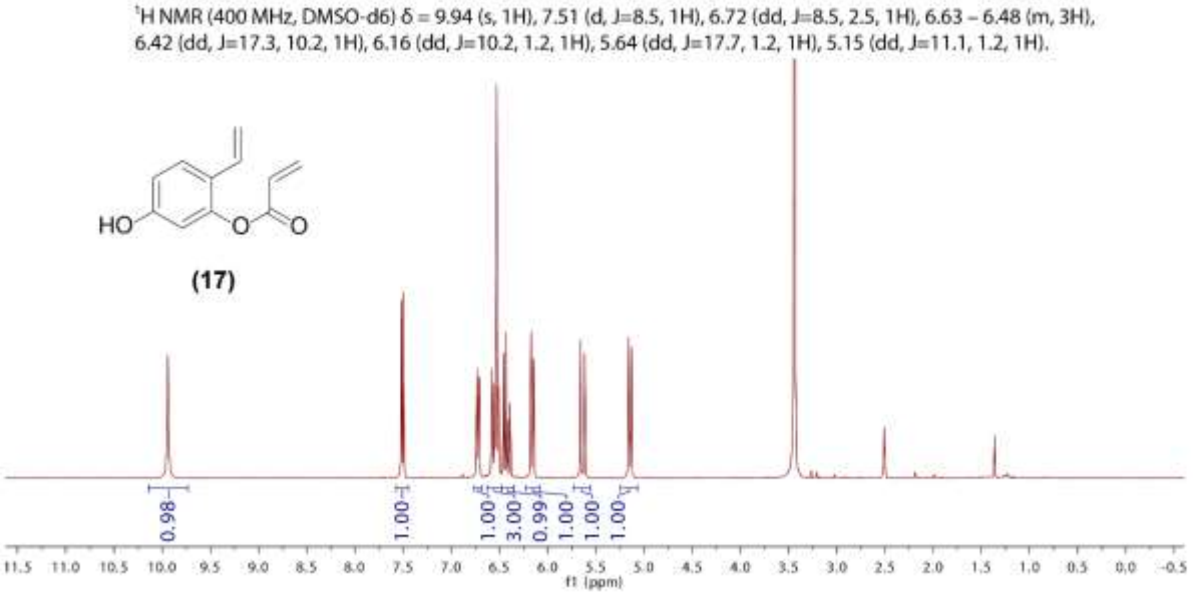

Figure S29. *^{1}H NMR spectrum of **17**.*

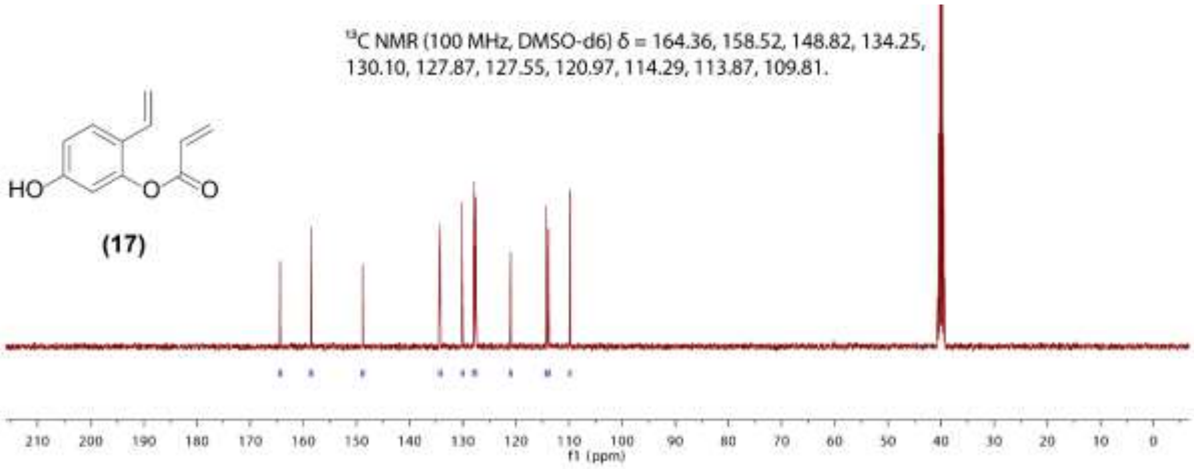

Figure S30. *^{13}C NMR spectrum of **17**.*

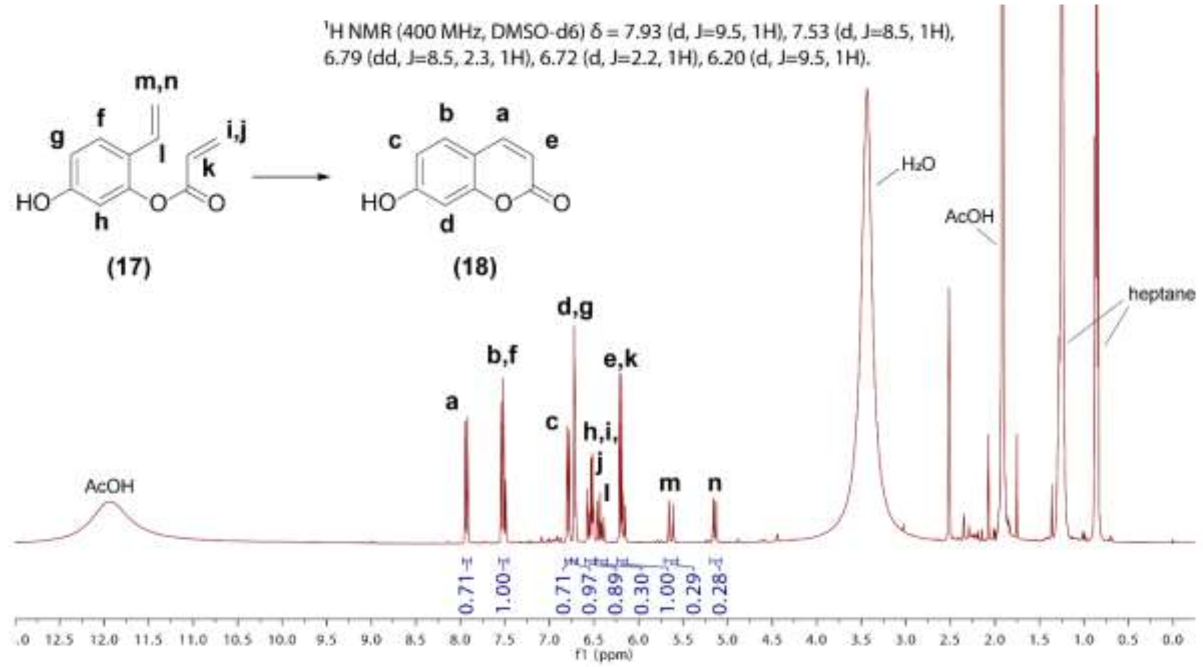

Figure S31. *^{1}H NMR spectrum of the crude mixture of the synthesis of umbelliferone from* ***17****.*

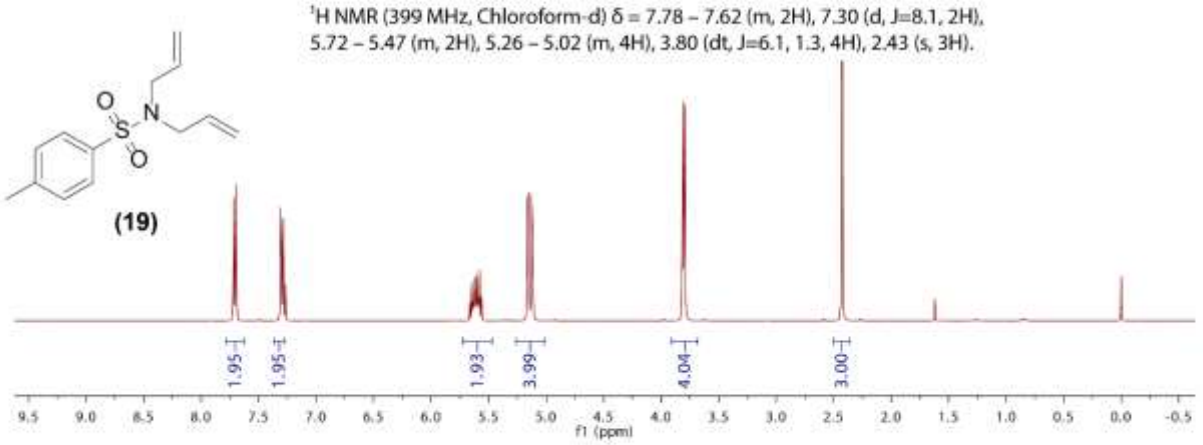

Figure S32. *^{1}H NMR spectrum of* ***19****.*

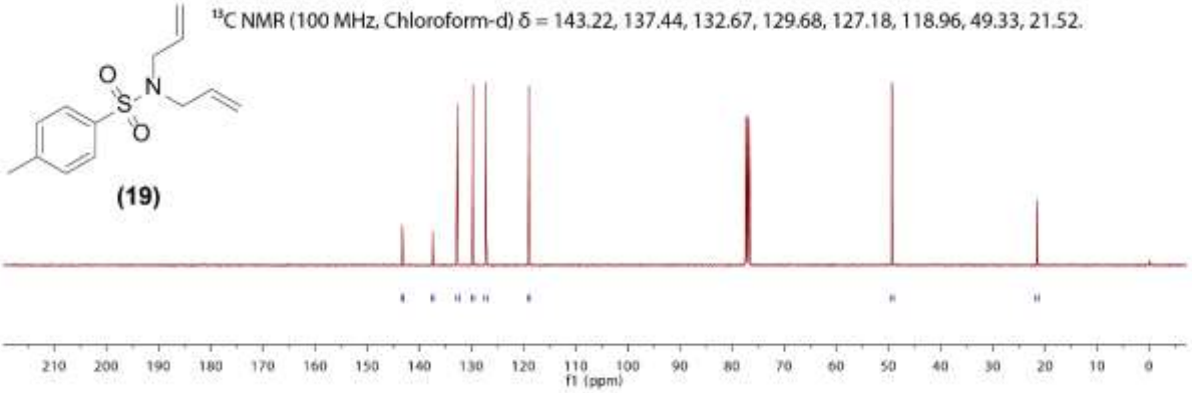

Figure S33. *^{13}C NMR spectrum of* ***19****.*

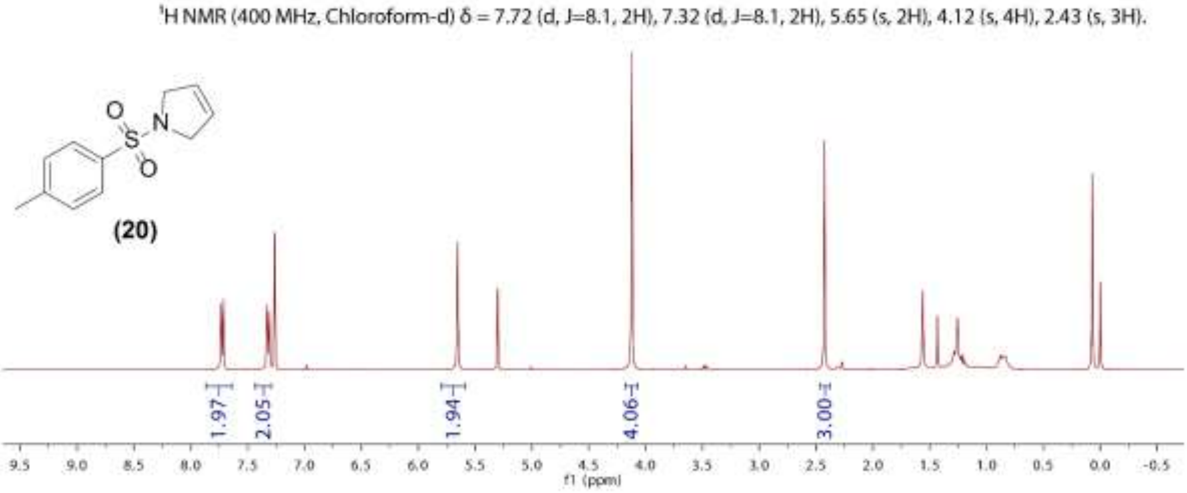

Figure S34. *^{1}H NMR spectrum of* ***20****.*

PEG$_{22}$-P(CL$_{38}$-*g*-TMC$_{36}$) (**6**)

PEG$_{22}$-P(CL$_{37}$-*g*-TMC$_{34}$-*g*-(TMC-N$_3$)$_2$) (**9**)

PEG$_{22}$-P(CL$_{37}$-*g*-TMC$_{34}$-*g*-(TMC-HGII)$_2$) (**12**)

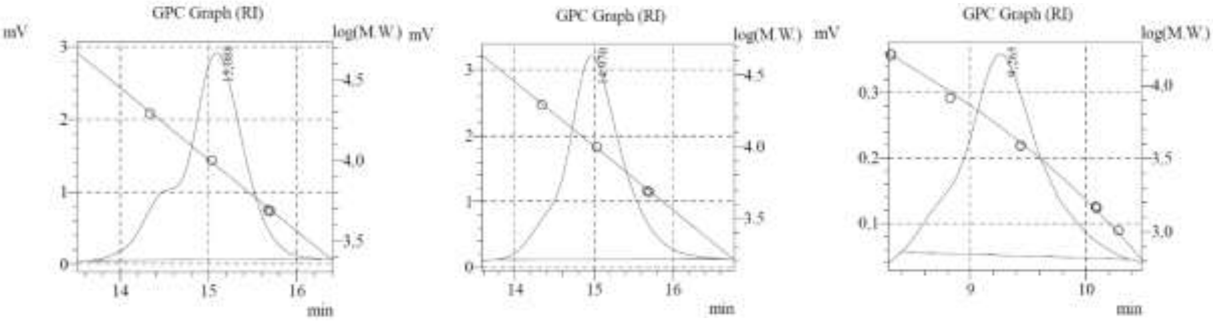

Figure S35. *GPC (THF) traces of copolymers* ***6*** *and* ***9****, GPC (DMF) trace of copolymer* ***12****.*

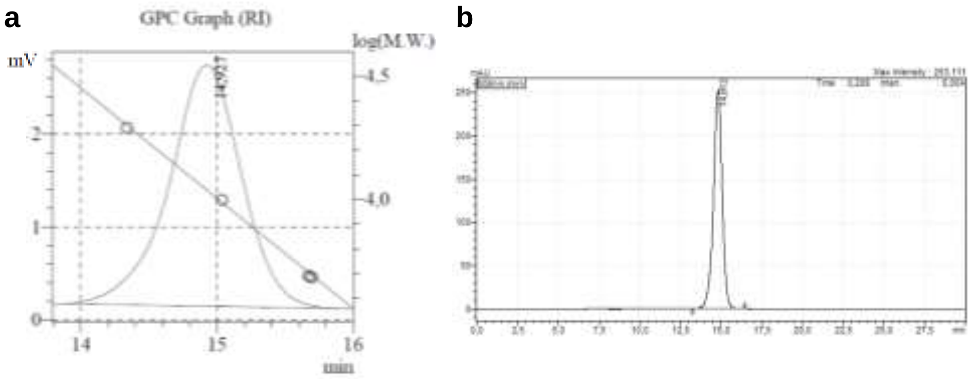

Figure S36. *GPC (THF) trace of Bodipy FL-block copolymer **13**. A) RI detector. B) PDA detector (506 nm).*[5]

	M_W	M_N	Đ
**6**	10977	9416	1.17
**9**	10866	9245	1.18
**13**	11651	10811	1.08

Table S2. *GPC analysis data of the different block copolymers. M_w and M_n values are based on a calibration curve of polystyrene standards.*

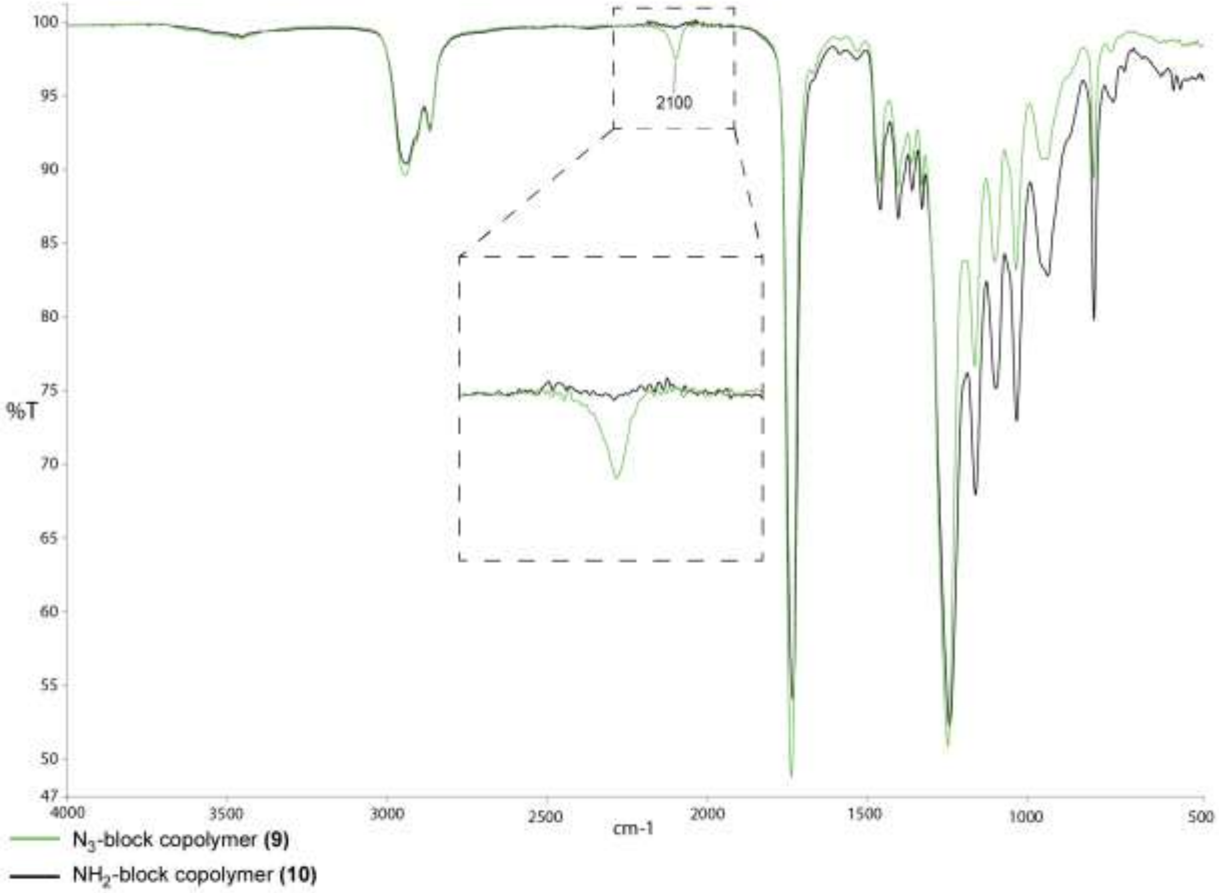

Figure S37. *IR spectra overlay of N_3-block copolymer (**9**) and NH_2-block copolymer (**10**).*

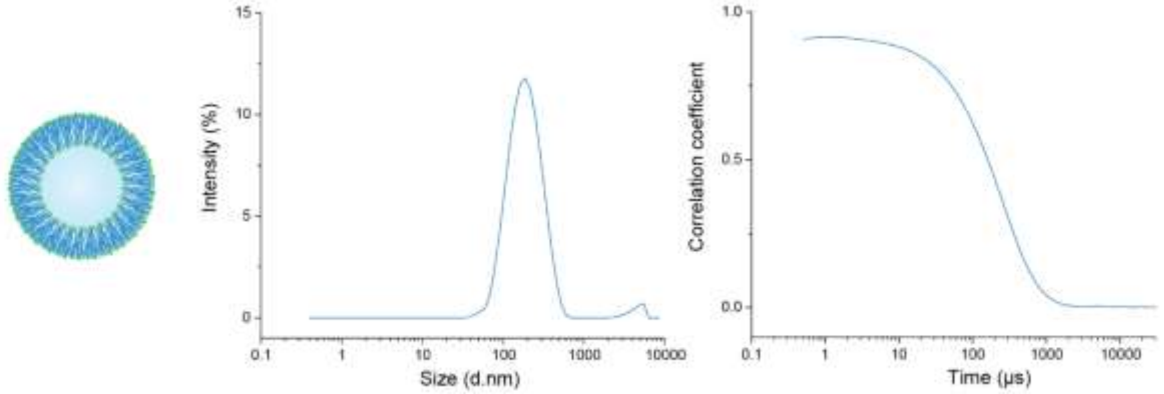

Figure S38. *Mean DLS intensity profile and correlogram of non-loaded polymersomes prepared from copolymer **6**, Z-avg=173 nm, PDI=0.22 (n = 3).*

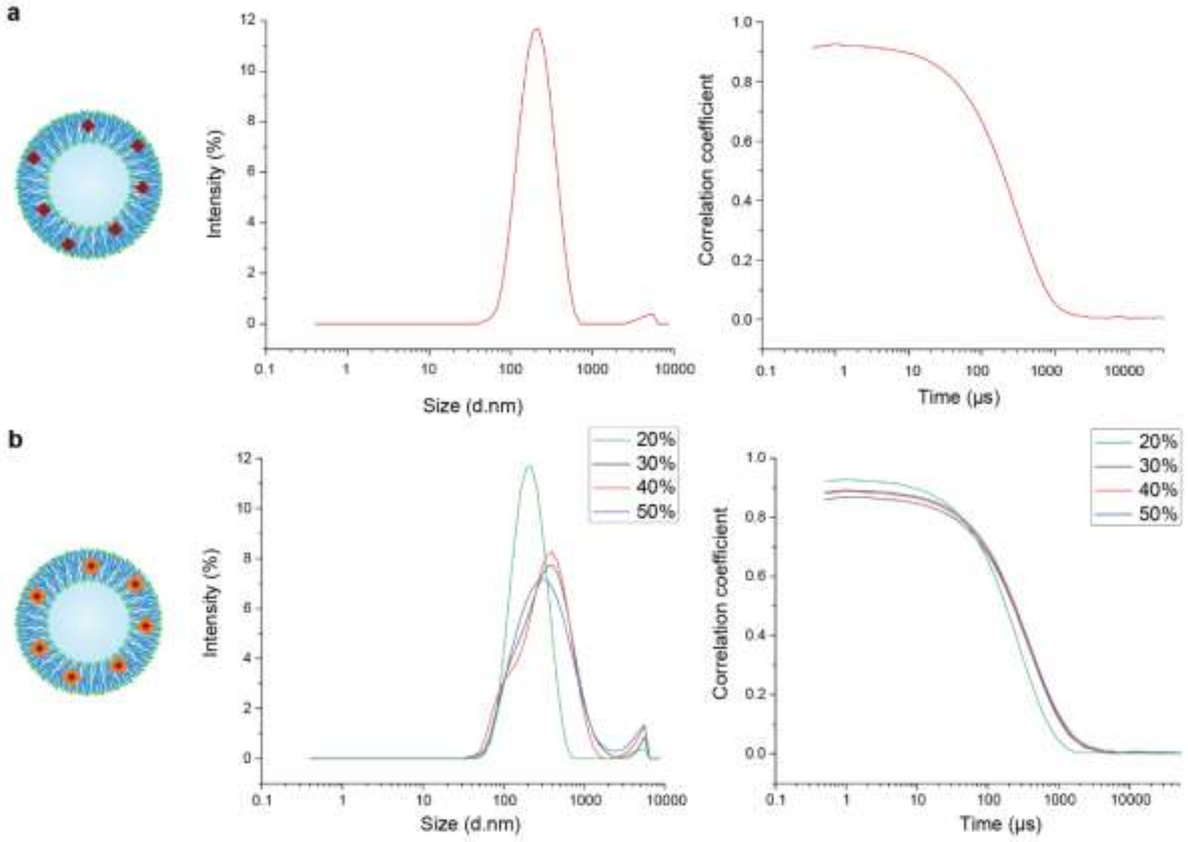

Figure S39. *Mean DLS intensity profiles and correlograms of HGII-loaded polymersomes (n = 3). A) Non-covalently loaded polymersomes. B) Polymersomes prepared from copolymer **6** together with 20-50 wt% of HGII-conjugated copolymer **12**.*

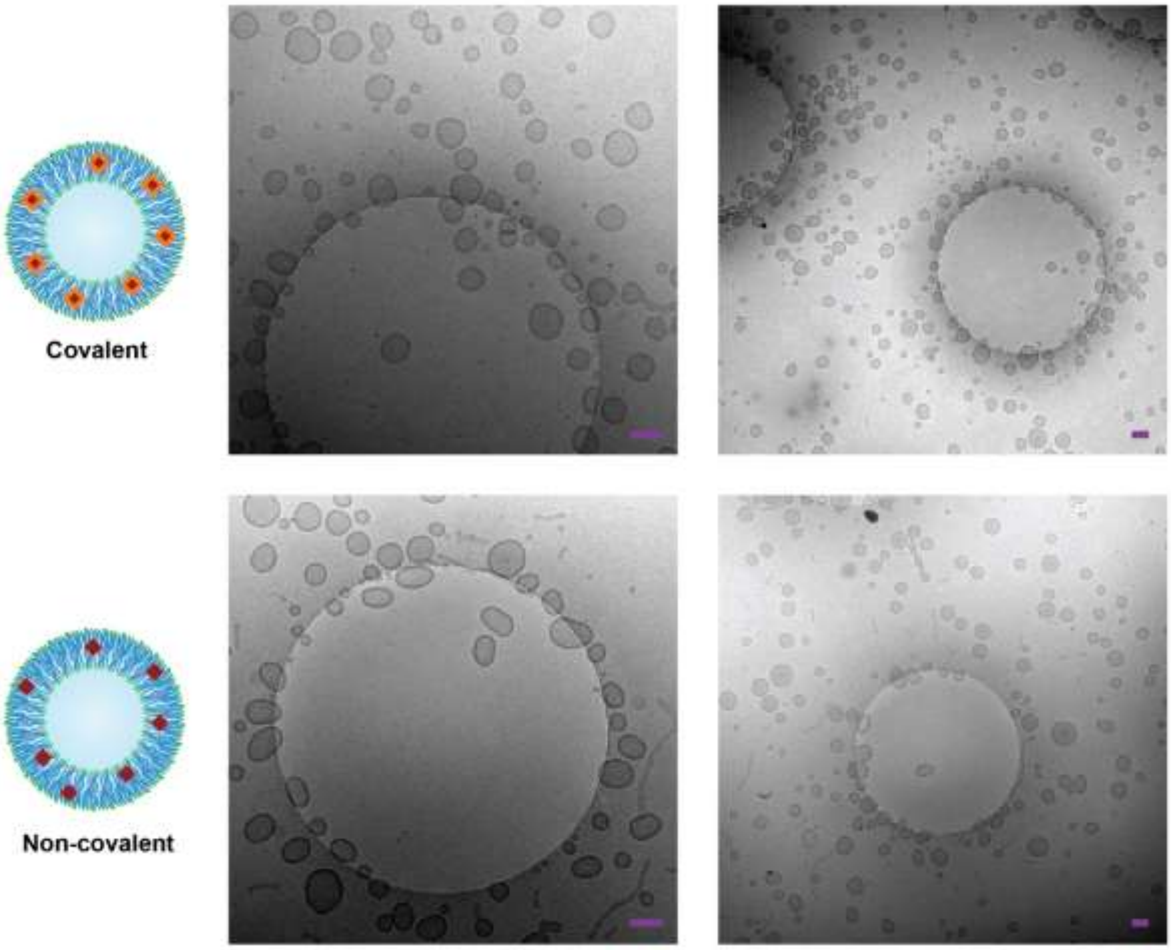

Figure S40. *Cryo-TEM images of nanoreactors either covalently (upper row) or non-covalently (lower row) loaded with HGII catalyst. Scalebar = 200 nm.*

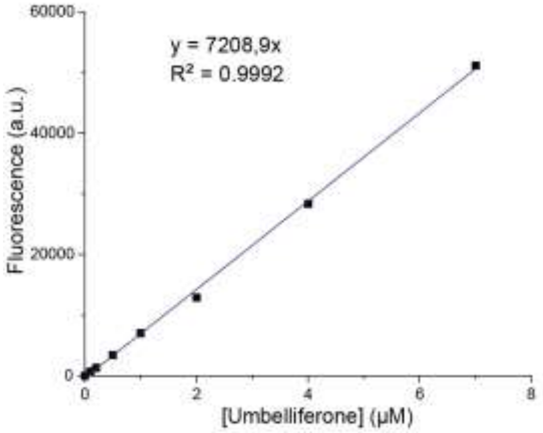

Figure S41. *Fluorescence calibration curve of umbelliferone.*

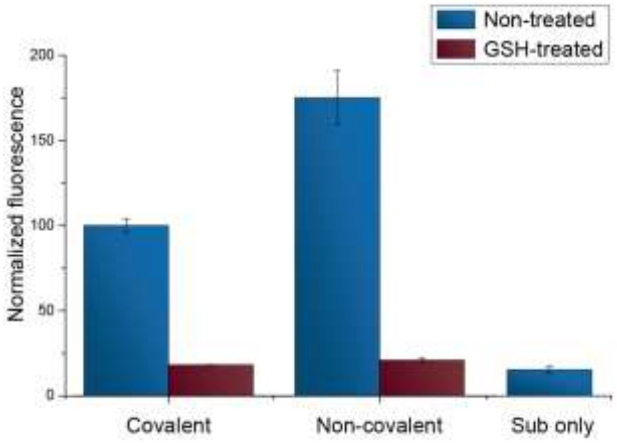

Figure S42. *Nanoreactor activity after treatment with glutathione. Nanoreactors were incubated for 16 hours with either PBS (non-treated) or glutathione in PBS (GSH-treated). Thereafter, the polymersomes were purified and incubated with 2 mM of substrate **17** for 20 hours at 37 °C. As negative control, 2 mM of **17** was incubated for 20 hours at 37 °C without the presence of nanoreactors. Then the resulting fluorescence was measured ($\lambda_{Ex}/\lambda_{Em}$ = 322 nm/440 nm) and normalized.*

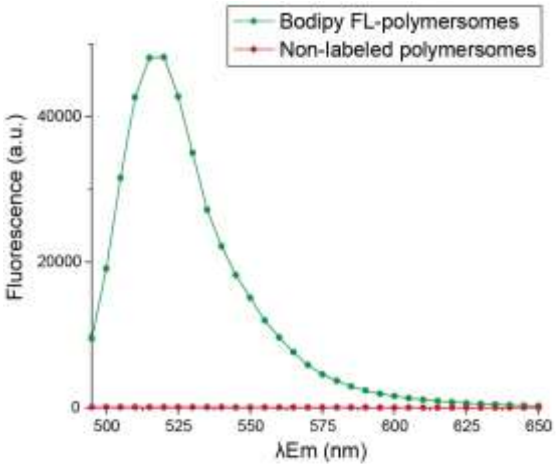

Figure S43. *Fluorescence emission scan overlay (λ_{Ex} = 450nm) of non-labeled polymersomes (1.0 mg/mL) and polymersomes (1.0 mg/mL) prepared from a mixture of copolymer (**6**) (75 wt%), HGII-conjugated copolymer (**12**) (20 wt%) and BODIPY FL-labeled copolymer (**13**) (5 wt%).*

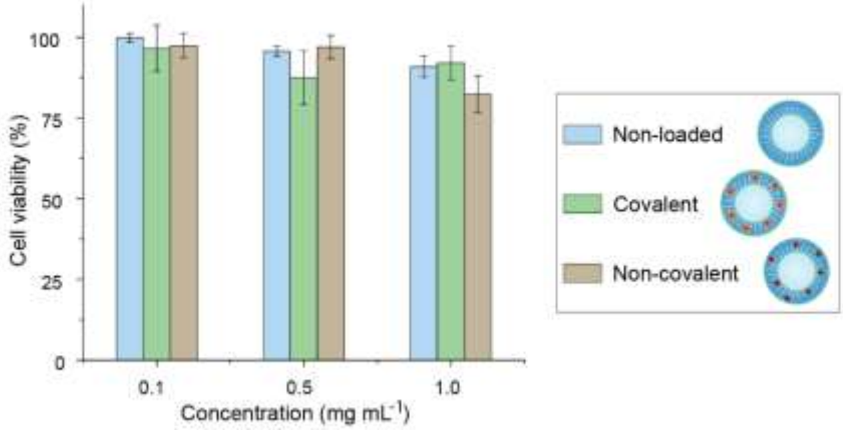

Figure S44. *Cell viability of polymersome-treated HeLa cells. HeLa cells were treated for 16 hours with either non-loaded polymersomes, polymersomes covalently loaded with catalyst or polymersomes which were non-covalently loaded (0.1-1 mg mL^{-1}). Their cell viability was subsequently analyzed via the MTT assay.*

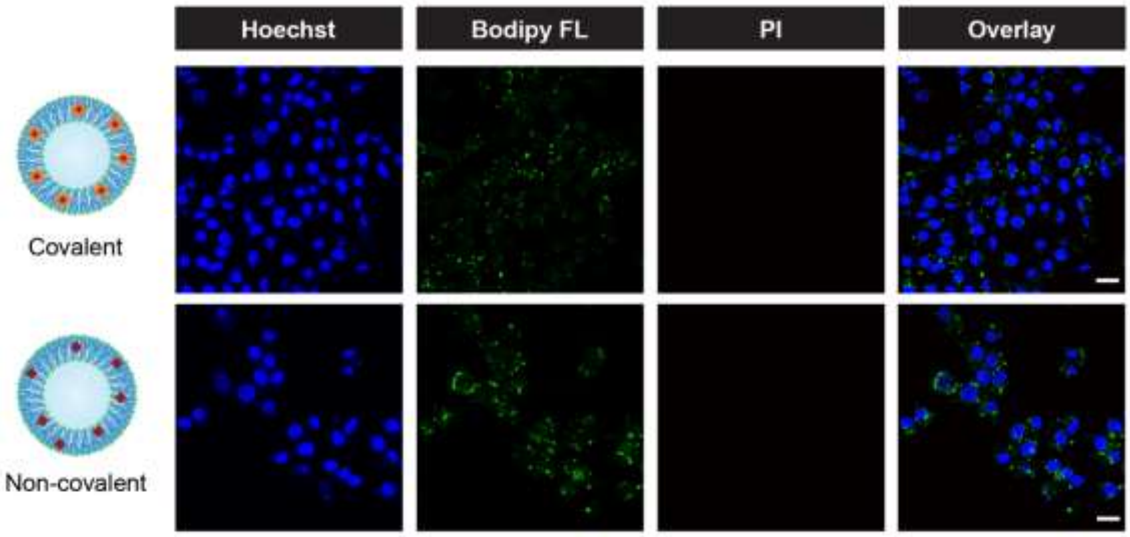

Figure S45. *Cell dead staining of nanoreactor-treated HeLa cells. HeLa cells were treated with Bodipy FL-labeled nanoreactors (1.0 mg mL^{-1}) for 16 hours, followed by staining with propidium iodide and Hoechst 33342 and imaged using CLSM. Scalebar = 25 µm.*

References

(1) Garber, S. B.; Kingsbury, J. S.; Gray, B. L.; Hoveyda, A. H. *J. Am. Chem. Soc.* **2000**, *122* (34), 8168–8179.

(2) van Oppen, L. M. P. E.; Abdelmohsen, L. K. E. A.; van Emst-de Vries, S. E.; Welzen, P. L. W.; Wilson, D. A.; Smeitink, J. A. M.; Koopman, W. J. H.; Brock, R.; Willems, P. H. G. M.; Williams, D. S.; van Hest, J. C. M. *ACS Cent. Sci.* **2018**, *4* (7), 917–928.

(3) Mason, A. F.; Yewdall, N. A.; Welzen, P. L. W.; Shao, J.; van Stevendaal, M.; van Hest, J. C. M.; Williams, D. S.; Abdelmohsen, L. K. E. A. *ACS Cent. Sci.* **2019**, *5* (8), 1360–1365.

(4) Sanders, D. P.; Coady, D. J.; Yasumoto, M.; Fujiwara, M.; Sardon, H.; Hedrick, J. L. *Polym. Chem.* **2014**, *5* (2), 327–329.

(5) Oerlemans, R. A. J. F.; Shao, J.; Huisman, S. G. A. M.; Li, Y.; Abdelmohsen, L. K. E. A.; van Hest, J. C. M. *Macromol. Rapid Commun.* **2023**, 2200904.

(6) Kapeller, D. C.; Bräse, S. *ACS Comb. Sci.* **2011**, *13* (5), 554–561.

(7) Kajetanowicz, A.; Chatterjee, A.; Reuter, R.; Ward, T. R. *Catal. Letters* **2014**, *144* (3), 373–379.

(8) So, C. M.; Kume, S.; Hayashi, T. *J. Am. Chem. Soc.* **2013**, *135* (30), 10990–10993.
